# Artificial intelligence vs. human coaches: examining the development of working alliance in a single session

**DOI:** 10.3389/fpsyg.2024.1364054

**Published:** 2025-04-15

**Authors:** Amber S. Barger

**Affiliations:** Teachers College, Columbia University, New York, NY, United States

**Keywords:** coaching, artificial intelligence, working alliance, coaching process, mixed methods, randomized controlled trial, wizard of oz

## Abstract

The collaborative relationship, or working alliance, between a client and their coach is a well-recognized factor that contributes to the effectiveness of coaching. The rise of artificial intelligence (AI) challenges us to explore whether human-to-human relationships can extend to AI, potentially reshaping the future of coaching. Our presumption that the skills of professional human coaches surpass AI in forging effective relationships stands untested — but can we really claim this advantage? The purpose of this study was to examine client perceptions of being coached by a simulated AI coach, who was embodied as a conversational vocal live-motion avatar, compared to client perceptions of partnering with a human coach. The mixed methods randomized controlled trial explored if and how client ratings of working alliance and the coaching process aligned between the two coach types in an alternative treatments design. Both treatment groups identified a personal goal to pursue and had one 60-min session guided by the CLEAR (contract, listen, explore, action, review) coaching model. Quantitative data were captured through surveys and qualitative input was captured through open-ended survey questions and debrief interviews. To sidestep the rapid obsolescence of technology, the study was engineered using the Wizard of Oz approach to facilitate an advanced AI coaching experience, with participants unknowingly interacting with expert human coaches. The aim was to glean insights into client reactions to a future, fully autonomous AI with the capabilities of a human coach. The results showed that participants built similar moderately high levels of working alliance with both coach types, with no significant difference between treatments. Qualitative themes indicated the client’s connection with their coach existed within the context of the study wherein the coach was a guide who used a variety of techniques to support the client to plan towards their goal. Overall, participants believed they were engaging with their assigned coach type, while the five professional coaches, acting as confederates, were blinded to their roles. Clients are willing to and appreciate building coaching partnerships with AI, which has both research and practical implications.

## Introduction

Interest and advancements in both the field of AI and professional coaching have experienced a marked upsurge in recent years. The disciplines have grown independently of one another, and now integrated opportunities between the two areas are emerging. In terms of AI, it has been around since the 1940s with fluctuations in starts and stops with investment and progression (Russell and Norvig, 2021). As of late 2022, the field of AI began an exciting new phase with the launch of generative AI systems that quickly gained popularity among the general public (Maslej et al., 2023). Within just 2 months following its public debut, ChatGPT attracted over 100 million monthly users, establishing a new global benchmark as the most rapidly expanding web application in history (OpenAI, 2023).

At the same time, the coaching profession has also been growing and changing. At the end of 2022, a study sponsored by the International Coaching Federation (ICF), the largest global professional association for coaches, showed a 54% growth in the number of coach practitioners since 2019 to approximately 109,200 individuals (International Coaching Federation, 2023). The same study found the total revenue from coaching services in 2022 was estimated at US$4.56 billion, a 60% increase from the 2019 estimate. Coaching industry leaders are already incorporating AI into their coaching products at companies like BetterUp, AIIR Consulting, Ezra, and CoachHub. AI applications are currently being developed to support specific portions of coaching practice, such as reinforcing new behaviors for clients between coaching sessions with Aiiron (AIIR Consulting, 2023), reviewing coach performance through AI-observed sessions with Ovida (2022), and supporting clients to set and make progress towards their goals with Coach Vici (2021). As Woody Woodward noted at the 2023 Coaching and Technology Summit, “the AI train has left the station” and the coaching industry is now differentiating its language between professional “human coaches” and AI coaches (Woodward, 2023).

Progress has been made in developing AIs for certain purposes, although the development of an AI that has the full capabilities of a human coach is still a long way away (Tambe et al., 2019). In a survey of prominent AI experts, they have starkly different views for when Artificial General Intelligence (AGI), or human-level AI, will be available; estimates range from 2029 to 2200 with an average estimated year of 2099 (Ford, 2018). As AI begins to replace or enhance some of the functions of a coach, a platform might someday be able to fully replace a human coach. Even in the absence of imminently available AGI that could possibly take the place of a human coach, it is worth exploring the wide applicability of AI within the coaching profession (Strong and Terblanche, 2020).

What if professional coaches could be replaced by AI? Former senior director of coaching at Google, the late David Peterson, proposed that, “In 10 years, 90% of what coaches do today will be done by artificial intelligence” (Boyatzis et al., 2022, p. 209). During a convening of 36 prominent coaching scholars of today, the group explored the future of coaching and called for more research on the role of various approaches to AI in effective coaching processes and outcomes (Boyatzis et al., 2022). Yet since that call to action almost 2 years ago, only a handful peer-reviewed original research studies have been published on AI coaching, which stem from the same primary researcher and mainly pertain to chatbots (Terblanche and Kidd, 2022; Terblanche et al., 2022a; Terblanche et al., 2022b; Terblanche et al., 2023a; Terblanche et al., 2023b).

## The present study

The present study examined two inter-related concepts – the coaching process, or model, by which the coaching session is organized and the working alliance, or partnership, that the client and coach form during the coaching session to help the client make progress towards their goal. These two concepts are intertwined because the coach is the facilitator of the relationship, and the relationship is formed through the way the coaching unfolds in the session and the use of the coach’s techniques to manage the conversation. The coaching process is an individualized approach that is tailored to the unique needs of each client in relation to their own situation and personal goals (Ely et al., 2010; Joo, 2005). Working alliance is defined as the measure of the client and coach’s active and shared commitment to purposeful collaboration within their relationship (O’Broin and Palmer, 2007, p. 305). In response to the identified gap in the literature about AI coaching, the purpose of this study was to empirically examine client perspectives when they were coached by a simulated form of autonomous AI, while comparing the same treatment intervention with participants coached by a human.

The working alliance between the coach and the client is one of the most important tools in effecting change and is a prerequisite for coaching effectiveness (Baron and Morin, 2009; Ely et al., 2010; Kampa-Kokesch and Anderson, 2001; Peterson, 2010). Working alliance has been shown as a key factor for impacting client outcomes from coaching, as indicated in dozens of studies (Graßmann and Schermuly, 2020). As well, Bickmore and Picard (2005) emphasize that in a human-computer working alliance, the element of trust becomes essential, particularly at times when clients are seeking to alter their behaviors or are required to exert substantial cognitive, emotional, or motivational effort. Several studies show early indication that affective bonds can be established within AI therapy and health coaching relationships, however, these are limited studies and the replicability of them is still unclear (Ellis-Brush, 2021). The literature indicates that human coaches form strong relationships with their clients; whereas AIs have a limited ability to do so.

The present mixed methods randomized controlled experiment had two research questions – one focused on the quantitative aspects and another focused on qualitative aspects. The primary research questions covered in in this paper were: (1) How do client ratings of working alliance align between clients coached by a simulated AI and clients coached by a human? and (2) What are clients’ perceptions of the coaching process and working alliance when participating in coaching delivered by a simulated AI or a human coach? The hypothesis in this study related to the first research question was that *clients who are coached by a human will have a greater working alliance than clients coached by a simulated AI*.

The type of coaching used in this study was non-directive whereby the coach supported the coachee, known as the client, to reflect upon their thoughts, feelings, and behavior to help the client to generate new insights, and then brainstorm how they wanted to take action towards reaching their personalized goal over the following weeks. It used an expert model of human coaching, meaning that the coaching was performed in the defined way that a trained, experienced human coach would execute the task (Terblanche, 2020). To explore how clients might respond to AI that mimics human coaching, this study used the Wizard of Oz (WOz) research technique that is common in the human-computer interaction field. Real professional human coaches served as the AI, while both the clients and coaches were blinded to this disguised treatment.

An extended study, not included in this paper, with a similar design is being conducted that includes the perceived value clients received from coaching, the change in perceived competence that clients had in relation to the goal they selected for themselves, and the extent to which clients made progress towards their chosen goal with comparisons to a control group.

## Literature

This section covers fundamental literature regarding the definition of coaching, the process of coaching, the human coach-client relationship, the definition of AI, expert systems, and the AI coach-client relationship to frame the statement of the problem for the present study.

### Definition of coaching

The ICF definition of coaching can be broadly applied to coaching of all types. It defines coaching as “partnering with clients in a thought-provoking and creative process that inspires them to maximize their personal and professional potential” (International Coaching Federation, 2018). Because it is non-directive and not domain-specific, professional coaching does not require formal expertise of the client’s subject matter by the coach. Coaching is about guiding the individual client to find their own solutions that will work for them in their unique life situation. It is goal-oriented and about unlocking potential and performance towards client-chosen outcomes.

By engaging in the coaching process with a professional coach, clients generally seek some type of change for themselves (Boyatzis et al., 2024). Clients come to coaching with their own unique background and circumstances, with varied aims to strive towards as a result of working with a coach. It is recognized that coaching works as one of “the most potent, versatile, and efficient” tools available for development (Peterson, 2010, p. 556), yet it is still an emerging discipline filled with contradictions about its preferred processes and optimal outcomes (Kauffman and Coutu, 2009). At its best, coaching is an individualized and adaptable phenomenon, which is one reason why it is difficult to measure. The coaching literature contains a wide variety of ways that coaching effectiveness and outcomes have been measured over the past few decades. Several meta-analyses and systematic reviews are available (Athanasopoulou and Dopson, 2018; Burt and Talati, 2017; Ely et al., 2010; Grover and Furnham, 2016; Jones et al., 2016; Sonesh et al., 2015; Theeboom et al., 2014), with the two recent ones by de Haan and Nilsson (2023) and Nicolau et al. (2023) that focused only on results from RCTs.

The literature generally aligns in that a key aim of coaching is for the coach to collaboratively foster the client’s personal or professional growth through a systematic, goal-oriented, and individualized process. The coaching process and the coach-client relationship are central components that constitute coaching and each of these are described next.

### Process of coaching

The way the coaching process unfolds in a session is a crucial component of the overall coaching dynamic. The coaching process is an individualized approach that has to be tailored to the unique needs of each client in relation to their own situation and their own personal goals (Joo, 2005; Ely et al., 2010). The process is goal-oriented; the client gains clarity about their current situation and their future and has a sense of accountability in terms of making progress toward their chosen goals (Peterson, 2010; Bartlett et al., 2014). An effective coach facilitates client learning within this process through a wide variety of competencies and techniques (Boyatzis et al., 2024; Maltbia et al., 2014). To facilitate change, clients are responsible for applying their new knowledge and skills in the real world outside of the coaching sessions themselves (Smith et al., 2009). Various perspectives of coaching exist, including cognitive behavioral coaching, mixed model/agile coaching, positive psychology strengths coaching, solution focused coaching, emotional intelligence coaching, systems-oriented coaching, goal setting coaching, gestalt/neuro-linguistic programming (NLP), and competency-based coaching (Parsloe and Leedham, 2022).

Coaching process can be facilitated by models to serve as navigational tools to remind coaches to include essential elements into the conversation. Ultimately, as with any model, strict adherence to the prescriptive sequence and structure is unnecessary. Instead, coaches navigate the parts of the model with flexibility, adapting to the client’s requirements and the flow of the dialogue. Even though longer-term coaching relationships seem to be the norm in the industry, one-time or laser coaching sessions on a single topic do happen. For these one-time, single sessions, a wide variety of coaching models exist to guide the coaching process. For the purposes of this study, the focus is on coaching models that could be used to facilitate a one-time session. GROW is the most widely known model of a coaching session structure, with 40.6 percent of coaching psychologists reporting having used it in a 2008–2009 survey conducted by Palmer (2011). GROW is an acronym for four interrelated phases within a coaching session: Goals, Reality, Options, and Wrap-up (Alexander, 2010). The GROW model was expanded upon by Downey (2003) into T-GROW by adding Topic at the beginning of the other four phases. OSKAR is a solution-focused session structure that stands for Outcome, Scaling, Knowhow and resources, Affirm and action, and Review (Jackson and McKergow, 2002). Additional models that have seven or more detailed steps include ACHIEVE (Dembkowski and Eldridge, 2003), PRACTICE (Palmer, 2007), and OUTCOMES (Mackintosh, 2005).

The CLEAR model was developed by Peter Hawkins in the 1980s and provides five stages to the process of coaching for a single session (Hawkins and Smith, 2013). The first stage is Contract, wherein the coach and client determine how they will partner together and define which goal the client wants to work towards. Even though the Contract stage normally happens at the beginning of a session, a coaching conversation is iterative, and the Contract can be revisited as new information is discovered throughout the session. The second stage of the CLEAR model is Listen. In the Listen stage, the coach supports the client to understand their situation at a deeper level by becoming aware of hidden assumptions and making new connections. The third stage is Explore, wherein the coach and client partner together to reflect and brainstorm potential options for moving towards the goal. Action is the fourth stage of the CLEAR model. The client decides upon a specific direction and commits to the initial action steps to get started. In the fifth and final stage, Review, the coach and client examine the how the contract was met and what the client has learned about themselves and their situation through the course of the session. In essence, the coaching process should unfold within a productive interpersonal relationship, one characterized by a mutual understanding and consensus on the objectives and tasks to be pursued (Adams, 2016).

### Coach–client relationship

Kampa-Kokesch and Anderson (2001) identified the relationship between the coach and the client to be one of the most important tools in effecting change. Having trust, rapport, and honest communication in the relationship (Ely et al., 2010; Peterson, 2010) is a prerequisite for coaching effectiveness (Baron and Morin, 2009). It is claimed that an effective coach should have the ability to establish strong, collaborative relationships (Bartlett et al., 2014). The coaching literature labels the relationship between coach and client as the working alliance (Bordin, 1979; Graßmann et al., 2019). The working alliance is a concept adopted into coaching research from the therapy and counseling fields (Bordin, 1979; Baron and Morin, 2009). It characterizes the relationship that is formed between two individuals in any helping relationship: the person who is seeking help and the person who is offering help. Bordin’s (1979) assumption was that the success of these helping relationships depends on the process by which the two individuals work together and the relation between the two. The working alliance includes the mutual agreement on goals and tasks between the helper and the person seeking help, along with the development of affective bonds. More specifically in coaching, the working alliance “reflects the quality of the client and coach’s engagement in collaborative, purposive work within the coaching relationship, and is jointly negotiated, and renegotiated throughout the coaching process over time” (O’Broin and Palmer, 2007, p. 305). The client’s perspective of the working alliance with their coach has shown to be more meaningful than the coach’s perspective because the client is the one who will be creating the change in their lives as a result of the coaching sessions (Graßmann et al., 2019).

A long-held assumption and finding in the coaching research is that the working alliance is a common success factor in coaching (Bluckert, 2005; O’Broin and Palmer, 2007; Vermeiden et al., 2022). A large number of studies exist that have analyzed the working alliance between clients and their coaches. McKenna and Davis (2009) found that the relationship factors between the coach and the client account for 30% of the success variance, in terms of being positive predictors of the client making change. Graßmann et al. (2019) found in a meta-analysis of 27 studies with *N* = 3,563 coaching processes that working alliance quality has a significant and consistent positive relationship with client coaching outcomes, including affective outcomes, cognitive outcomes, and individual-level results outcomes with varying effects sizes. Additionally, the systematic review by Graßmann and Schermuly (2020) identified a clear connection between working alliance and coaching process, the central coaching component in the previous section.

However, recent studies indicate that the quality of the working alliance, as measured by the Working Alliance Inventory (WAI) (Horvath and Greenberg, 1989), might not have so much to do with the efforts of the coach and client collaborating together, but the client’s “general tendency and ability to form satisfying relationships with others” as a trait (de Haan et al., 2020; Molyn et al., 2022, p. 221). de Haan et al. (2020) proposed that scores from the WAI, the most commonly used working alliance measure in coaching research, are generally stable over the length of a coaching relationship. While a positive rapport with the coach does matter, it scarcely affects the progressive changes brought about by subsequent coaching sessions. In a recent meta-analysis of only randomized controlled trials de Haan and Nilsson (2023) found that the number of sessions, or length of the coaching relationship, does not seem to matter much as it relates to gaining higher or better outcomes for clients after a certain point (Nicolau et al., 2023). It further confirmed previous literature, originally from psychotherapy (Stiles et al., 2015), that proposed the phenomenon of coregulation, wherein coaches and clients are able to adjust to maximize their time together in order to achieve their chosen goals (de Haan and Nilsson, 2023; Sonesh et al., 2015; Theeboom et al., 2014).

### Definition of AI

The AI field is focused on theoretically understanding, but also ‘*building* intelligent entities – machines that can compute how to act effectively and safely in a wide variety of novel situations’ (Russell and Norvig, 2021, p. 1). In the real-world, AI is a collection of technologies, such as natural language processing, computer vision, robotics, virtual agents, and machine learning (Bughin and Hazan, 2017). Since AI was conceptualized in the 1940s and 1950s, the long-term vision of it has remained the same to this day: to have an AI platform be able to think, learn, and perform like a human (McCarthy, 1958; Shane, 2019; Russell and Norvig, 2021). In the short-term, the industry has yet to reach this goal, and it has been necessary to define simpler gradations of AI.

Young et al. (2019) define three shades of AI, listed from least intelligent to most intelligent: assisted, augmented, and autonomous. The most intelligent is autonomous intelligence, an AI system that can “adapt to different situations and can act autonomously without human assistance” (p. 10). At the other end of the scale is the least intelligent form of AI, assisted intelligence that consists of “AI systems that assist humans in making decisions or taking actions; hardwired systems that do not learn from their interactions” (p. 10). Between autonomous and assisted intelligence lies augmented intelligence, which is a type of AI that can “augment human decision making and continuously learn from their interactions with humans and the environment” (p. 10). Most AI systems available today are either assisted intelligence or augmented intelligence, with very few approaching autonomous intelligence.

Alternative models of AI maturity exist, including a distinction between weak AI and strong AI (Searle, 1980); the concepts of Artificial Narrow Intelligence (ANI), Artificial General Intelligence (AGI), and Artificial Super Intelligence (ASI) (Russell and Norvig, 2021); and a continuum from bot, basic AI, advanced AI to super AI (Clutterbuck, 2022). The gold standard of autonomous intelligence approaches AGI, where a true thinking machine replicates human intelligence or better (Ford, 2018). More recently, conversational AI agents (e.g., chatbots, voice assistants) have emerged that use speech or text to mimic human interaction to simulate conversations (Kulkarni et al., 2019; de Cock et al., 2020). Conversational AI can be visually represented by avatars, “digital entities with anthropomorphic appearance, controlled by a human or software, that are able to interact” (Miao et al., 2022, p. 67). The generative AI platforms that have become increasingly available to the public over the past year can be categorized as augmented intelligence, because of the need to have a human in the loop of creation while the AI learns from its interactions and feedback from a human.

### Expert systems

The idea of expert systems (or knowledge-intensive systems) that try to emulate the decision-making process of a human through the use of if-than rules (Russell and Norvig, 2021), was developed throughout the 1970s. According to Lucas and Van Der Gaag (1991) expert systems are “systems which are capable of offering solutions to specific problems in a given domain or which are able to give advice, both in a way and at a level comparable to that of experts in the field” (p. 1). To create a specific expert system, like an AI professional coach, it can be done by referencing chosen knowledge sources, such as expert human coaches, coaching textbooks or training manuals, and recordings of exemplar coaching sessions. If an AI coach were to have an expert system design, it means that the system would be modeled after how an expert human coach would execute the task of coaching. Terblanche (2020), a preeminent scholar of AI coaching research, suggests the use of the established coaching principles (e.g., strong coach-client relationships, goal-oriented process) to create a foundation for the design of AI coaches. Again, the most intelligent form of AI is autonomous, wherein the AI can act independently while adapting well within different novel situations and learn on its own (Young et al., 2019). No such autonomous technology exists now in the coaching field and is likely to not arrive for some time (Russell and Norvig, 2021). In the meantime, less intelligent forms of AI, the assisted and augmented types, can be designed using expert systems of human professional coaching. In the past few years several conceptual models of AI coaching have been proposed, with four of those conceptual models briefly summarized next in chronological order of publication.

Terblanche (2020) presented a novel framework, Designing AI Coach (DAIC), that uses four principles generated from expert human coaching systems for the design of assisted or augmented AI. The first principle is for the AI to build a strong relationship with the coaching client by displaying certain attributes like empathy, transparency, and predictability. The second principle is for the AI to be designed with evidence-based coaching practices that have been shown to work well specifically in the context of coaching. The third principle is for the AI to be designed with both coaching ethics (i.e., fostering client autonomy, providing clarity on stakeholder responsibilities) and data science ethics (i.e., upholding data security and privacy, reducing embedded bias). The fourth principle is for AI coaches to be designed to have a specific narrow focus, such as starting a career transition or developing work-life balance, due to the inability for current AI capabilities to perform well on a wide variety of conversation topics, like a regular human coach could. Terblanche (2020) had a specific recommendation to use the DAIC for conversational agents or chatbots.

Graßmann and Schermuly (2020) conceptually analyzed to what extent AI (i.e., assisted, augmented) could guide clients through the systemic PRACTICE coaching process (Palmer, 2007) via Brynjolfsson and Mitchell’s (2017) AI evaluation criteria. The PRACTICE model consists of seven steps: (1) Problem identification, (2) Realistic, relevant goals developed, (3) Alternative solutions generated, (4) Consideration of consequences, (5) Target most feasible solution(s), (6) Implementation of Chosen solution(s), and (7) Evaluation. The first step, problem identification, proves difficult for an assisted or augmented AI to perform because AI cannot read between the lines and understand clients’ intentions. Additionally, in the development of specific goals, AI cannot offer feedback on chosen goals or identify gaps that clients had not thought of yet. According to Graßmann and Schermuly’s (2021) assessment, AI does have the ability to perform the remaining six steps of the PRACTICE model relatively well as long as certain considerations are taken into account in the AI coach design process. With this analysis it is important to consider that the PRACTICE model is focused on goal setting and constructing solutions, and does not explicitly incorporate other approaches to coaching that might be more reflective and less structured.

Clutterbuck (2022) published a perspective on how basic forms of AI (i.e., assisted) compare to human coaches and also how basic AI and human coaches could partner together. He did this analysis in alignment to a list of six coaching tasks aligned to the GROW model, six common skills of coaches (e.g., listening, rapport building), and four attributes of coaches (e.g., compassion, courage). As an example, with the task of establishing the coaching purpose and goals, the human coach alone would “work with context and values before agreeing to goals,” the AI coach alone would “focus on the goal and routes to achieving it” and be “unable to work easily with evolving goals” (p. 376). With the human coach and AI coach partnering together, there would be “deeper exploration of context and purpose” and the ability to “look beyond initial goals” (p. 376). Clutterbuck suggested that by integrating human coaches and AI coaches it could provide more benefit than either stand-alone option by “raising awareness by extracting clarity and purpose from complexity, in order to exercise better judgment and create more positive outcomes” (2022, p. 374).

Duhan et al. (2023) proposed an AI coaching model that links detailed coaching elements to conversational AI strategies. First, the authors mapped the ICF coaching core competencies to conversational AI design strategies and suggestions for how AI can support human coaches based upon prior research. The model was derived from several definitions of expert human coaching with a focus on three parts – establishing the coach-client partnership, facilitating the coaching process, and enhancing client outcomes. Then, the coaching model was mapped to specific conversational AI design and development strategies (Martin, 2019; Martin, 2023), including defining the AI coach persona, designing basic AI conversation aspects, and enhancing the AI conversation design to be more complex. Next, the authors proposed desirable attributes of AI coaches and conversational AI design strategies to specific coaching process. For example, with the coaching process technique of active listening the desired attribute of the AI coach is to “exhibit understanding for better [client] engagement,” therefore the design strategy is to have the AI “repeat, summarize, confirm” (Duhan et al., 2023, p. 181). This flexible model can be further extended to include additional coaching approaches (e.g., solution-focused, cognitive-behavioral) and coaching techniques (e.g., action planning) in order to adapt to the needs of specific AI coach personas.

These are four examples of conceptual models for AI coaching that have been proposed within the past few years. The models are based upon the concept of expert systems – using what works from human expert coaches and translating that into the design of AI.

### AI coach–client relationship

The relationship, or working alliance, between the coach and the client is one of the most important tools in effecting change and is a prerequisite for coaching effectiveness (Baron and Morin, 2009; Ely et al., 2010; Kampa-Kokesch and Anderson, 2001; Peterson, 2010). According to Bickmore and Picard (2005), trust within a human-computer working alliance is crucial when clients desire behavior change and when they need to offer significant cognitive, emotional, or motivational effort. For decades, the human-computer interaction field has studied the relational dynamics between humans and technology, with recent advancements seeing a significant shift towards integrating AI into the research. Not many studies currently exist that analyze an AI coach-client relationship, or working alliance, in the context of professional coaching (Terblanche et al., 2024; Mai et al., 2022). Therefore, a wider view of studies from other modalities, such as counseling, motivational interviewing, and health coaching, have been reviewed.

It is difficult to compare results across these studies because they are different in modality and focus of participant change. Studies have used modalities of interaction such as digital chatbots that are text-based, others have used digital avatars that are anthropomorphic to look like humans, and still others have used types of physical robots to interact with the human research participants. The focus of change ranges from reducing exam anxiety (Mai et al., 2022), facilitating goal progression (Terblanche et al., 2022a), reducing symptoms of depression (Fitzpatrick et al., 2017), to improving self-resilience (Ellis-Brush, 2021), and more. To layer in more complexity, these studies have used technologies at different states of maturity ranging from scenario-based hypothetical user reviews to technologies that are currently available today to future-state technologies that are portrayed to users through WOz experiments. Overall, it is safe to say that the findings across the studies are somewhat inconsistent with one another. In some cases, individuals have built a positive relationship with the technology interface, and in others they have not, with reasons that vary based upon the study’s specifics, such as the modality of interaction, the focus of change, and the population in the study.

Several of the sampled studies show that participants did develop a relationship with their AI or technology-enabled coach. Mai et al. (2022) conducted a study to compare engineering students’ use of two different types of chatbots to facilitate self-reflection – one that prompted users to click and the other that prompted users to write. It found that participants using either of the chatbot types rated the working alliance with the AI coach as medium to high. In another study conducted by Mai et al. (2021) that used an interaction script via a WOz experiment, it assessed different types of disclosure behaviors from a chatbot on the client relationship. It was found that information disclosure by the chatbot generated more self-disclosure and rapport among student participants on the topic of exam anxiety. This study found that students were open and transparent with the chatbot.

Another study on working alliance in the field of coaching was conducted by Terblanche et al. (2024). This novel study did not directly measure the working alliance between a client and an AI coach. Instead, it qualitatively measured working alliance between a client and a human coach when an AI chatbot coach named Vidi was used in between the coaching sessions that the client had with their human coach. In the hybrid coaching framework, Vidi served as a tool to facilitate client reflection, monitor progress towards objectives, and strategize for upcoming coaching sessions. Interview responses indicated that clients were at ease disclosing information to the chatbot, which they found to be helpful in advancing toward their objectives. Although Vidi was praised for its convenience and utility, it was also perceived as lacking a personal touch. Coaches were also asked their opinion about the chatbot after seeing a demonstration of it. The coaches has mixed reviews of Vidi – citing nervousness that it might interfere with their own relationship with their client, while also saying that it could be useful for select clients to support them in making progress towards their goals in between coaching sessions.

From other modalities outside of coaching, one study found that incorporating relational skills such as empathy and social dialogue into the bot user interface significantly improved working alliance and user engagement, as evidenced by a 30-day intervention with subjects to help them foster physical health activity (Bickmore and Picard, 2005). In another physical activity study, Bickmore et al. (2010) discovered that participants did establish a working alliance with their AI coach. In the healthcare field, Lucas et al. (2014) found that in clinical interviews, participants disclosed more information and felt less judged by their interviewer when they believed their virtual human interviewer was artificially intelligent compared to if the participant thought their virtual human interviewer was being operated by a human. Several studies have shown that when AI systems use human rapport-building behaviors, such as sharing humanlike emotions, having a human name, or displaying facial expressions that participants wanted to keep engaging with them (Lisetti et al., 2013; Park et al., 2023; Portela and Granell-Canut, 2017; Seo et al., 2018).

Another concept related to the coach-client relationship is the notion of amount of usage, or dosage of the treatment. Terblanche and Cilliers (2020) proposed that trust between humans and AI may not be as important as the amount of application use. Their study examined technology acceptance constructs (e.g., facilitating conditions, perceived risk) through the lens of individuals who had recently participated in at least one coaching conversation with a goal attainment coaching chatbot, Vici. The results showed that participant performance expectations, or the extent to which they believed the application would perform well to help them, had the most influence on the intent of participants to use the chatbot (Terblanche and Cilliers, 2020). This could mean that regardless of a strong working alliance, as long as the AI coach is useful, then it could provide benefits to users. In another study of real-world users of the Wysa application, the individuals who engaged with the application most reported a significantly higher average improvement score in self-reported symptoms of depression compared with the low users group (Inkster et al., 2018). When using Woebot, participants who used the chatbot significantly reduced their symptoms of depression over the study period, while those in the control group (who did not use the chatbot as much) did not reduce their symptoms of depression (Fitzpatrick et al., 2017). The concept of usage is especially relevant as AI coaches have the potential to be available to support clients at all hours of the day, unlike human coaches.

Other studies have shown that working alliance was not developed between research participants and their AI coach. A recent study conducted using the Wysa chatbot application (Ellis-Brush, 2021), found that a working alliance did not develop between the client and AI application, yet the majority of participants were still able to reach their goal to improve their self-resilience. In the study, participants from a financial company who had a high degree of computer usage engaged with a chatbot, visualized as a penguin, on their phone over the course of 8 weeks using cognitive behavioral therapy (CBT) techniques. Through both quantitative measures and qualitative interviews, Ellis-Brush (2021) found that that users of the Wysa application had a transactional interaction with the chatbot and were apathetic as to whether a working relationship did develop. This study points to the notion that maybe working alliance is not as important in human-to-computer coaching relationships as it is in human-to-human coaching relationships.

Another study from the healthcare field used a scenario method to ask participants if they would trust a human doctor or AI system more when receiving a prescription medication recommendation after a medical examination (Yokoi et al., 2021). The study found that even when the AI system performed just as a human doctor would, that participants did not trust it as much as the human doctor and preferred the human doctor to give the prescription recommendation, even if it was the exact same. This evident skepticism toward AI, despite its functional equivalence to human doctors, underscores a broader ambivalence in human and AI interactions. Other concepts in the literature related to working alliance with technology-enabled coaches are engagement (Bickmore et al., 2010), trust (Yokoi et al., 2021), acceptance (Lisetti et al., 2013), self-disclosure (Zhang and Rau, 2022), and usability (Fitzpatrick et al., 2017).

Overall, there are mixed findings regarding the affective bond and working alliance between clients and AI coaches that could be beneficial to investigate further. These studies show early indication that affective bonds have the possibility to be established within coaching relationships, however, there are a limited number of studies and the replicability of them is still unclear (Ellis-Brush, 2021).

## Methods

To take on the challenge set by Boyatzis et al. (2022), this study adds to the coaching research by using a simulated autonomous AI who can do what the assisted and augmented AIs cannot – imitate human behavior and perform as an expert human coach (Young et al., 2019). It is important to note that autonomous AIs that behave as humans are rare to find (e.g., AlphaGo and its successors), and are currently not available in the coaching field. With the limits of technology constantly changing month by month, the researcher did not want to design an AI that would be outdated in a short amount of time. *Instead, this study was carefully designed to facilitate participants’ belief and perception that they were being coached by an AI, when in fact it was expert professional human coaches behind the scenes operating with their real coaching skills.*

For participants to fully experience an autonomous AI, one that is able to fully act as a human, real human coaches were used as confederates in this study using the WOz technique. This study was meant to capture insights related to the future-state of AI in the coaching field and how clients would respond if and when an AI could, in fact, act exactly like a human coach would. Research in human-computer interaction is conducted in a wide variety of ways with participants, including using co-design workshops, individual or group interviews, interactive prototype testing, WOz procedures, and established commercial systems (Clark et al., 2019; Sadasivan et al., 2023). A WOz is an experimental research procedure where participants interact with a computer system they believe to be autonomous, but which is actually being operated or partially operated by an unseen human being, much like the “wizard” behind the curtain in the story of “The Wizard of Oz” (Dahlback et al., 1993). WOz experimental studies are “proactively deceptive” to influence participants in certain ways to believe they are interacting with an intelligent system, whereas, in reality, a human operator controls the responses to explore human-computer interactions and system design (Porcheron et al., 2021, p. 243). WOz techniques are valuable for exploring human-computer interactions, particularly when the technology is not yet available or is too costly to implement in an experimental phase.

**Figure 1:**
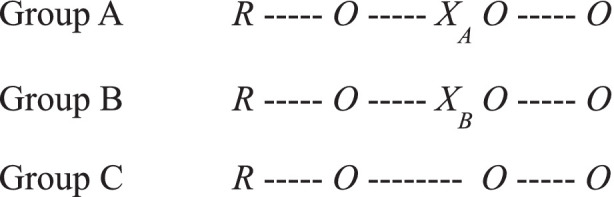
Notation of alternative-treatments design. The symbol *R* indicates random assignment. *O* represents an observation or measurement recorded on an instrument. *X* represents an exposure of a group to an experimental variable (Campbell and Stanley, 1963, p. 6).

The study was designed as a mixed methods randomized controlled trial (RCT) with participants randomly assigned to one of two treatment groups or a control group (see Figure 1; Creswell and Creswell, 2018; Creswell and Plano Clark, 2018). Group A received the innovative treatment (X_A_), being coached by a simulated AI; While Group B received a more standard treatment (X_B_), being coached by a human. Group C is the control group and did not receive treatment during the experiment’s data collection time period, and is part of the extended study not included in this paper.

Each participant set a goal they wanted to achieve and agreed to make progress towards that personal goal over the course of 1 month. Those in the two treatment groups received one 60-min coaching session to help them gain clarity on their goal and design action steps to take towards their goal. A survey was conducted after the coaching session was complete. A couple of weeks after the coaching session half of the individuals were randomly assigned to participate in a debrief interview about their experience. A mixed methods design was used with an intent to gain a more complete understanding of the phenomena by bringing together the results of the qualitative and quantitative data analysis on this topic that has been explored very little to date.

### Research setting

The setting where this study took place was in a private university based in the southwestern part of the United States. The university has a leader development institute (Institute) that supports the entire population of graduate and undergraduate students across all schools of the university. To date, the Institute has provided coaching programs for up to 35% of the student population. Therefore, the general student population is familiar with professional coaching.

It is relevant to note the time period within which this study was conducted: March through June 2023. McKinsey & Company (2023b) called the year 2023 as *Generative AI’s Breakout Year* to describe its explosive growth during this time. In November 2022 OpenAI first released ChatGPT, an AI-powered large language model that could create human-like text based on context and past prompts (OpenAI, 2022). Soon after in March 2023 GPT-4 was released that drastically improved upon the already astounding capabilities of the original GPT-3.5 (OpenAI, 2023). During this time, business and education society was abuzz with AI-hype – with non-technical individuals not fully understanding what was or was not possible with the AI tools (Budhwar et al., 2023). This hype in the professional sphere and daily media headlines about the advances in AI likely influenced participant perspectives about this study.

### Participants

To be eligible to participate in this research study, participants needed to (1) have been enrolled as a graduate student at the university, (2) have voluntarily signed up for and completed a one-on-one leadership coaching program in the past, (3) have a real goal they were ready to make progress towards over the next month, and (4) have not been trained a coach themselves via the completion of a 60+ hour coach training program. This group, in terms of education profiles and career progressions, was selected from the broader university population (Shadish et al., 2002). Individuals were excluded from the study if they did not meet the aforementioned criteria.

The target sample size was developed in consultation with the Institute’s measurement team, led by a social psychologist who had been assessing the effectiveness of coaching programs at the university. The Institute used similar psychological measures with the same population in a variety of scenarios for several years. Based on this experience, a target recruitment sample size was determined to consider the number of people needed to show statistically significant results, while also anticipating usual attrition rates. To get statistically significant results in each group, each needed a minimum of 20 participants with an ideal target of 25 people per group. When the ideal number of participants per group was met and those individuals had completed all requirements, the study was concluded.

**Figure 2:**
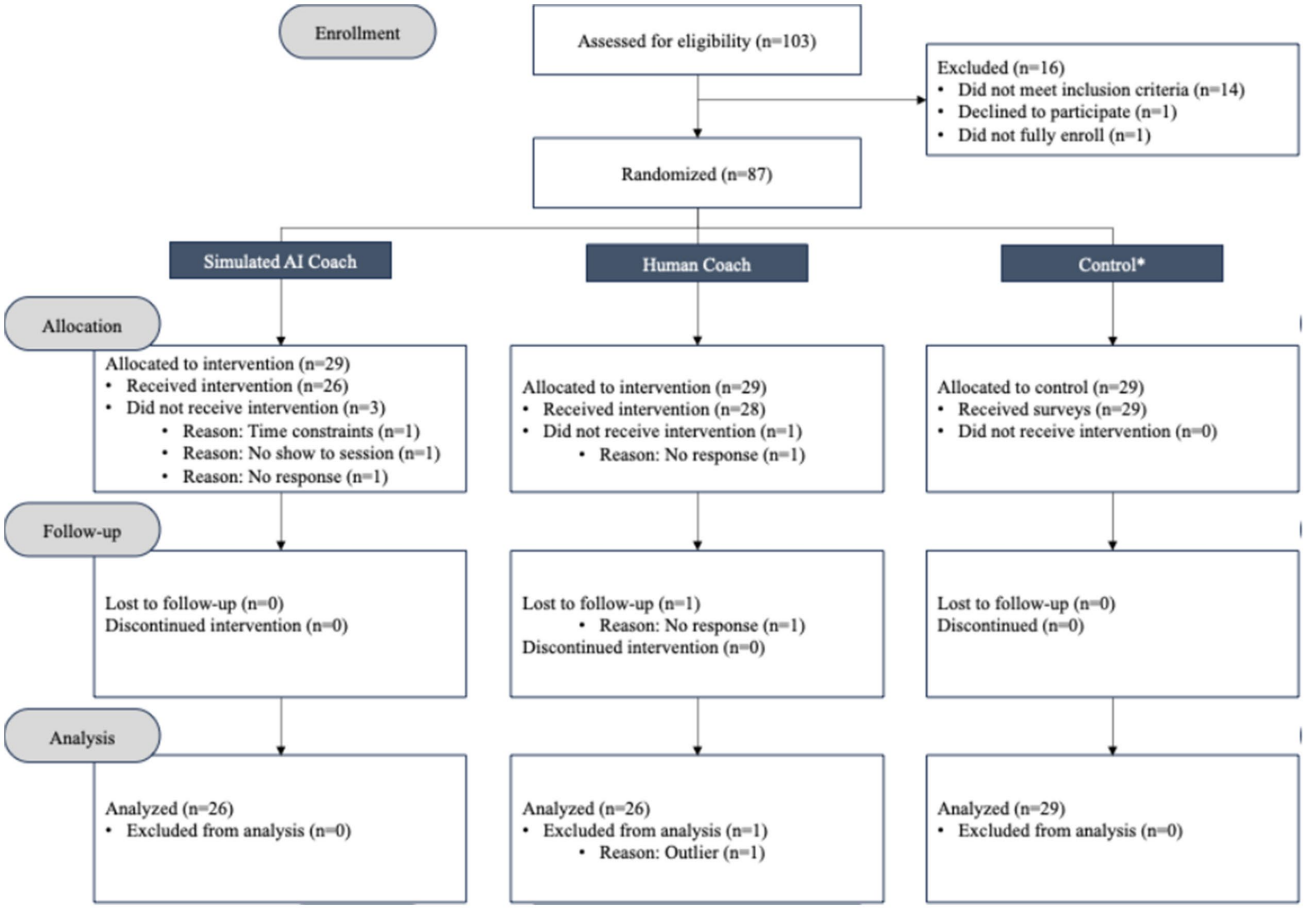
Participant flow from enrollment through analysis. The control group is out of scope for this report and is part of an extended study.

To recruit the optimal number of participants, all the individuals who met the inclusion criteria were invited to the study. To target the graduate students who met the inclusion criteria, a variety of recruitment activities were conducted including sending individualized emails, giving presentations at student government meetings, posting fliers on bulletin boards in busy buildings on campus, and sending announcements within the graduate student association weekly newsletter. As noted in Figure 2, a total of 52 individuals enrolled and fully completed the study, with 26 individuals who were randomly assigned to be coached by the simulated AI coach and 26 individuals who were randomly assigned to be coached by the professional human coach. As part of an extended study, a control group is included to assess different research questions that are out of scope for this paper.

### Description of the intervention

After meeting the eligibility criteria, participants gave their sociodemographic information and chose a goal they were motivated to work towards over the next month. Thereafter, the researcher performed the randomization procedures to place individuals in one of three groups. Of those who were selected to be coached by either the simulated AI (X_A_) or the human (X_B_), half of those were randomly selected to participate in a debrief interview. Each participant was sent an individualized email that contained specific information regarding their assigned next steps in the study, including the explicit assignment of an AI coach or professionally trained human coach.

The main component of the intervention was a 60-min coaching session between the client and coach. Immediately after completing the coaching session, each participant received the link to a survey. The survey included both quantitative measures and qualitative questions related to the experience in the session. Two weeks after completing the coaching session, those who were randomly chosen then participated in the 45-min semi-structured debrief interview.

#### Description of coach role

The success of the coaching intervention relied heavily on the professional coaches who chose to be part of the study. The five professional coaches who were part of the study each held the Professional Certified Coach credential from the International Coaching Federation. Each coach had at least a decade of experience as a coach, as well as at least 5 years of experience coaching this specific population of university students. The coaches operated within the study as confederates who had specific roles in the experiment to control for certain manipulations (Leis and Reinerman-Jones, 2015). To prepare the coaches to use the study-specific coaching model within the experimental design protocols, the primary researcher facilitated several onboarding activities, including a one-on-one orientation session, two group training sessions, reference guides, and pilot practice sessions. The CLEAR coaching model was the one that was used in this study for the 60-min coaching session (Hawkins and Smith, 2013). According to the coaches, in all 52 of the coaching sessions all five of the parts of the CLEAR model were covered, resulting in a 100% adherence rate.

To facilitate the coaches being blind to which condition they were assigned, many techniques were used in a thoughtful, integrated manner. To avoid bias, confederates were as naïve as possible to the research questions and measures of the experiment and the condition that they were participating in Kuhlen and Brennan (2013). The researcher organized the sessions and set the context with clients in a way that reduced client inquiries about the AI in the session itself. The technology and equipment set-up was organized by the researcher in a way that the coaches could not visually recognize which condition they were assigned in that session.

In about 7% of coaching sessions, the coaches mentioned that they might have known which condition they were in and came to the insight towards the end of the session. During four of the coaching sessions, the coach thought they knew which condition they were in based upon either something the client said or a technology error. For the most part, these occurred towards the very end of a coaching session, therefore these samples have been kept in the analysis.

#### Coaching treatment

At its essence the study sought to understand client reactions to an AI coach who performed in ways akin to a human coach, and then compare that to a real human coach group, and in the extended study compared to a control group. In the between-subjects experiment, each participant was only tested in one condition. The two treatments – the simulated AI coach and human coach – were designed to have only one difference between them. The one difference was either the client’s perception that their coach was an AI or the perception that their coach was a human. Other than that, all other particulars of the treatment remained the same. *For all participants and in all sessions, the coaching was delivered by a trained, experienced professional coach who is human.*

A difference between the simulated AI and human coach treatments was the way the participant experienced their coach visually and auditorily during the session. Both types of sessions were conducted via the Zoom platform. With the human coach treatment, the coach was visualized as themselves on the screen with a plain grey background. With the simulated AI coach treatment, an Avatar that looked like the coach was used with a plain grey background. With the incorporation of the Animaze software, the avatar moved dynamically on the screen to match the coach’s facial expressions and non-verbal body language. The use of an avatar to take the place of the human visual on the screen was necessary to uphold the deception. The choice of avatar type was intentional. It had human-like features because research shows that individuals perceive this type of avatar as credible and engage with the avatar in similar human-to-human social rules (Holzwarth et al., 2006; Nass and Moon, 2000; Wang et al., 2007; Westerman et al., 2015). Additionally, the avatar design chosen had a simplistic anthropomorphic appearance because a realistic anthropomorphic appearance is shown to have an uncanny valley, or creepiness, effect on people (Miao et al., 2022). Research has shown that intelligent avatars that have cognitive and emotional intelligence are especially effective for complex, relational transactions involving sensitive personal information (Lucas et al., 2017). For this study, it was most effective for people to interact with intelligent avatars with a simplistic anthropomorphic appearance.

**Figure 3:**
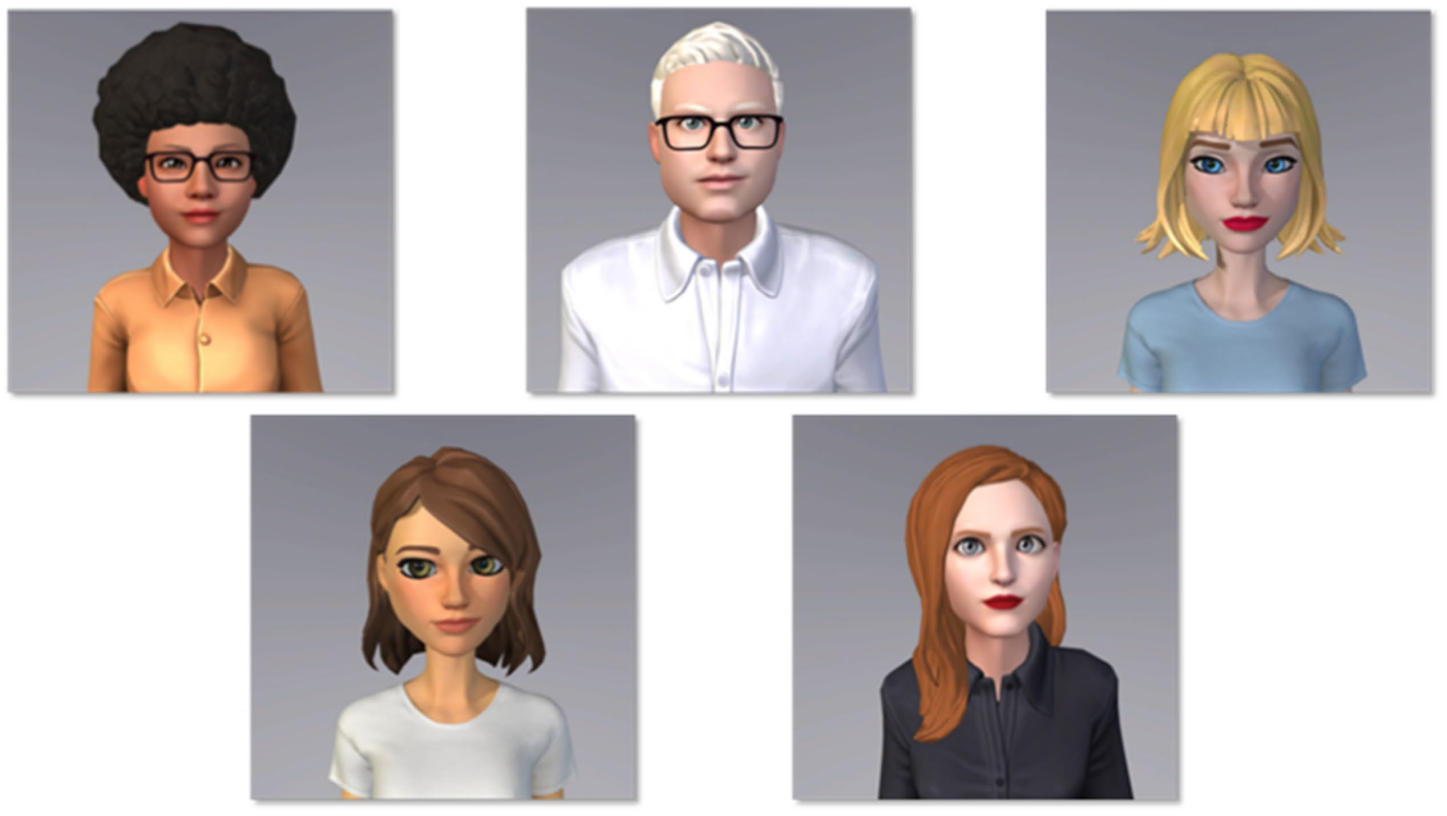
Avatars of the five coaches created using Ready Player Me software, https://readyplayer.me/.

The primary researcher created an avatar that looked like each of the coaches themselves using the Ready Player Me software (see Figure 3). As much as possible, skin color, eye color, hair color, and hair style were made to match each coach from real life. Special accessories like glasses and make-up colors were matched, too. Matching the avatar to the real-life coach was done on purpose in order to reduce unintentional effects of clients making misaligned assumptions about the coach based upon the visual representation on the screen.

Another difference between the simulated AI coach and the human coach was the voice of the coach. With the human coach treatment, the regular voice of each coach was captured through the Zoom microphone and broadcasted to the participant. With the simulated AI coach treatment, a voice distortion software, VoiceMod, was used to change the sound of each coach’s voice when it was broadcasted through to the participant. This design choice was necessary in order to make the simulated AI coach more believable that it was a real AI. The software made the voice sound slightly robotic. Without this design feature, it is doubtful that deception for the simulated AI coach would have worked. Again, the coach could not detect the change in their own voice, only the client could.

### Measures and analysis

To answer the research questions, this study used both quantitative and qualitative data collection methods and analyses, as well as a newly constructed Believability Index to understand to what extent participants believed the treatment they were assigned.

#### Quantitative

The Working Alliance Inventory (WAI) measures client perception of the quality of the coaching relationship, or the client and coach’s engagement in collaborative, purposive work (Bordin, 1979; Hatcher and Barends, 1996). The WAI was first published by Horvath and Greenberg (1989) to measure the relationship between therapist and client. The original version has 36 items in three subscales of 12 items each, rated on a 7-point Likert scale. The traditional inventory assesses three key aspects of the alliance: (a) agreement on the tasks of coaching, (b) agreement on the goals of coaching and (c) development of an affective bond. After examining the complete 36-item WAI, Tracey and Kokotovic (1989) developed a 12-item short form of the WAI (WAI-S). The high correlations found between the three dimensions of the WAI have led many researchers to use the average WAI score as a measure of the alliance (Hatcher and Gillaspy, 2006). Hatcher and Gillaspy (2006) developed a revised short-form for the working alliance inventory (WAI-SR) that would more clearly distinguish Bordin’s task, goal, and bonds dimensions.

The WAI-SR was collected from individuals who participated in the coaching session from both the simulated AI and human coach groups directly after the session occurred. The working alliance inventory had a very high level of internal consistency, as determined by a Cronbach’s alpha of 0.934 using the sample from this study. Each subscale also had a high level of internal consistency, agreement on the tasks of coaching (*α* = 0.807), agreement on goals of coaching (*α* = 0.836), and development of an affective bond (*α* = 0.871). An independent-samples *t*-test was conducted to evaluate whether there were statistically significant differences in the mean working alliance scores between the two distinct treatment groups.

#### Qualitative

Qualitative data were collected in two ways through open-ended survey questions responses and through semi-structured debrief interviews. Several questions were posed to participants to elicit responses related to the coaching process and working alliance. To analyze the qualitative data, the researcher employed the method of maximum variability sampling as a strategy to construct a comprehensive codebook from the debrief interviews (Saldana, 2021). This method enabled the researcher to intentionally select six participants, three from the human coach treatment group and three from the simulated AI coach treatment group, that exhibited maximum diversity in perspectives within the scope of the study. Subsequently, the researcher applied the constant comparison analysis technique to systematically examine and categorize the data (Saldana, 2021). This iterative process involved comparing new data with previously coded segments, identifying emergent themes, and refining the codebook accordingly. The final codebook incorporated themes and sub-codes from both the human coach treatment group and simulated AI coach treatment group in one view. At the same time, the researcher further enriched the qualitative analysis by incorporating the *in vivo* coding technique to directly use participants’ own words to label interim sub-codes and themes within the data. This approach was chosen because it preserves the authenticity and context of participants’ expressions, adding depth to the findings (Saldana, 2021). Each transcript was coded using the final codebook, which resulted in a count of sub-codes and illustrative quotes per sub-code shown in the next section. The use of maximum variability sampling, constant comparison analysis, and *in vivo* coding collectively strengthened the rigor of the qualitative research, resulting in a contextually nuanced understanding of the client perspectives about the coaching sessions and their experiences.

#### Believability index

WOz studies need to be thoughtfully constructed and presented to participants with a believable fiction (White and Lutters, 2003). Believable fiction refers to the carefully constructed illusion that participants are interacting with a fully functioning autonomous system. The fiction must be convincing enough for participants to behave as if the system were real, thus allowing researchers to observe genuine reactions to the technology being tested. The “believability” of the system is crucial, as it ensures that the data collected on user behavior, interaction, and satisfaction are valid within the scope of the study, even though the underlying technology might not yet be capable of such autonomous operation.

WOz studies rely on this believable fiction to gain insights into how users might interact with future technologies and to guide the design and development of these systems before they actually exist. To check whether and to what extent participants believed their coach to be an AI or a human, the three-part manipulation check was turned into an index. All three parts were included in the survey. Rather than reply on one manipulation check question, and to detect as much suspicion as possible, the three-part manipulation check gave ample opportunity for participants to be forthcoming about any suspicion.

Part A was an open-ended manipulation check question asking who the coach was, modeled after several other studies (Ho, 2018; Lucas et al., 2014; Zadro et al., 2004; Branigan et al., 2011). The question was, *Who did you have a conversation with in this study?* This was intentionally an open-ended question, rather than closed-ended. A close-ended question could prompt participants to alter, post-hoc, their perceptions of the coach. Part B was a funneled debriefing (Bargh and Chartrand, 2000; Ho, 2018) that was meant to uncover additional suspicion that might not have been revealed in Part A. The open-ended questions in the funneled debriefing were: *What do you think the purpose of this study was?, Was there anything unusual about the study? If so, what was it?, Was there anything unusual about your partner? If so, what was it?* The researcher coded the answers to these open-ended questions on a 6-point scale: Human, Probably Human, Likely Human, Likely AI, Probably AI, and AI.

Part C is a two-item set of questions that has been adapted from Ashktorab et al. (2020). It measured the extent to which participants perceived their coach to be an AI or a human. The two items were: *I believe I was interacting with an AI* and *I believe I was interacting with a human*. This study used Likert-type agreement anchors ranging from 1 = *Strongly Disagree* to 6 = *Strongly Agree* with no mid-point choice option. The Believability Index is the combination of these three items: (1) Researcher’s code of qualitative answers, (2) Answer to AI belief survey question, and (3) Answer to human belief survey question. For the applicable index (i.e., AI, human) one of the survey items was reverse-coded to match it.

Additionally, in the coach report, the coach indicated whether the client asked about the technology, avatar, or AI in the session. This allowed for additional analysis and consideration regarding levels of believability.

### Ethical considerations

This section details the ethical considerations devised for this study wherein some participants were deceived into believing they were receiving a coaching session from an AI, when in fact it was a professional human coach who was facilitating the conversation. The study received full approval from a university’s Institutional Review Board (IRB) and followed all requirements to protect human subjects. Ethical considerations included informed consent, privacy and confidentiality, potential harm and discomfort, fair treatment and selection, and feedback to participants. First, before each person enrolled in the study it was important to obtain informed consent from each participant. This consent was not merely a signature on a form but involved a process wherein participants were educated about the study’s area of focus, procedures, potential risks, and benefits.

To uphold participants’ rights to privacy and confidentiality, all personal identifiers were removed or anonymized during data analysis and reporting. Pseudonyms were given to participants. Data storage followed strict security protocols, with access limited strictly to the researcher. Any quotes or case studies drawn from qualitative data were carefully selected to ensure the anonymity of participants.

To address potential harm and discomfort to participants, the research team partnered with the university’s counseling center with trained counselors available throughout the duration of the study in case participants might have needed this support. For establishing fair treatment and selection, the randomized nature of the experiment required attention to ensure that participants were selected without any bias. Randomization was executed using a computerized system, ensuring that every participant had an equal opportunity to be placed in one of the groups. These reduced potential biases related to education program, gender, race, or other characteristics that could influence the outcomes.

At the end of the study, after all the data was collected from each participant, a debrief letter was emailed to inform participants about the true nature of their assignment. The letter explained the reasons why the deception was necessary, a description of the preliminary results, and a list of references to access if they wanted to learn more. As stakeholders in the research process, this step ensured participants were informed about the results of the research they contributed to in order to foster respect and reciprocity.

## Results

This section covers the details of the individuals who participated in the study, as well as the quantitative results and qualitative findings that are underpinned by the believability index.

### Participants

The participants in this study were all graduate school students at the same university who each had previous experience with coaching. The following sociodemographic data was captured: education program, gender identity, race/ethnic identity, and age. To align with the CONSORT guidelines for reporting parallel group randomized experiments, the sociodemographic information is shown for each of the treatment groups—human coach and simulated AI coach (Table 1).

**Table 1:** Sociodemographic characteristics of participants by group.

	Human Coach	AI Coach		
Sample characteristics	*N*	%	*N*	%
Education program				
MBA	13	50.0%	14	53.8%
Master	2	7.7%	4	15.4%
PhD	11	42.3%	8	30.8%
Gender identity				
Female	13	50.0%	15	57.7%
Male	13	40.0%	11	42.3%
Non-binary	0	0.0%	0	0.0%
Race				
Asian	6	23.1%	13	50.0%
Black or African American	2	7.7%	3	11.5%
Hispanic	8	30.8%	2	7.7%
White	8	23.1%	5	19.2%
Other	1	3.8%	1	3.8%
Two or more races	2	7.7%	2	7.7%
Prefer not to say	1	3.8%	0	0.0%
Age				
18–22 years old	1	3.8%	0	0.0%
23–26 years old	7	26.9%	4	15.4%
27–30 years old	9	34.6%	9	34.6%
31–34 years old	7	26.9%	7	26.9%
35–38 years old	1	3.8%	5	19.2%
39–42 years old	1	3.8%	1	3.8%

The total number of individuals in the human coach sample is 26. The total number of individuals in the AI coach sample is 26.

### Quantitative results

**Figure 4:**
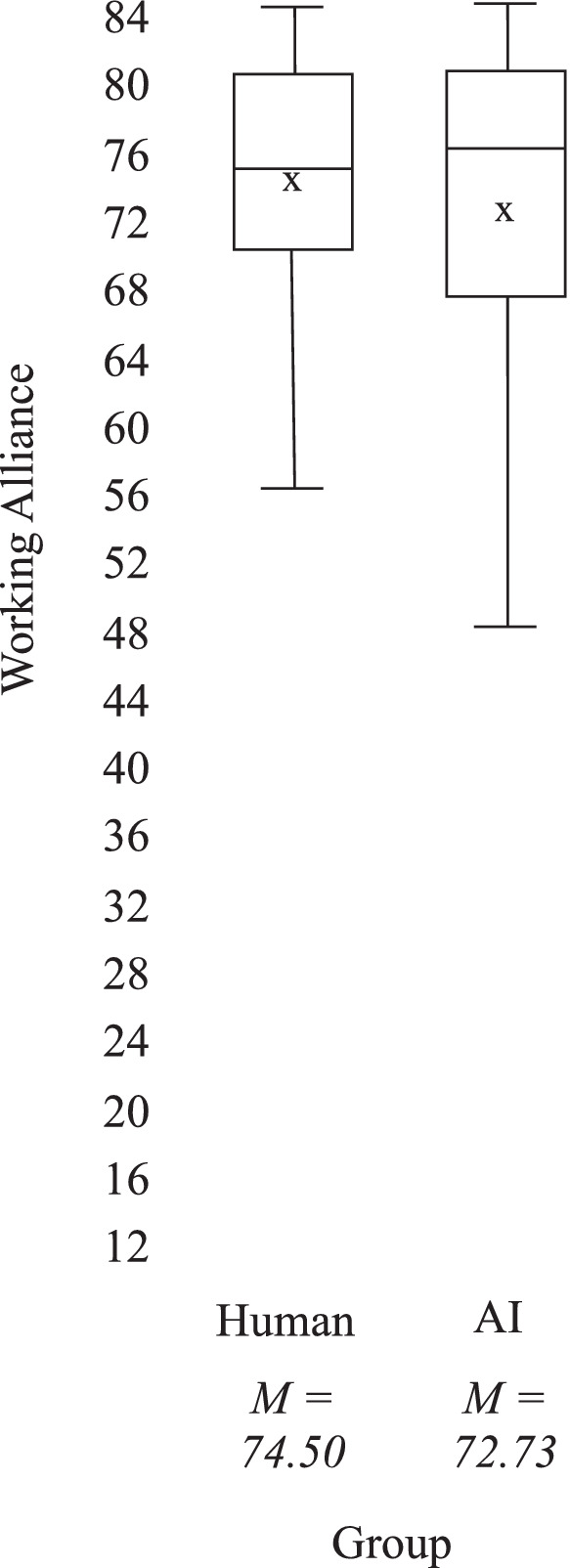
Working alliance ranges in human vs. simulated AI coach treatment groups. The bottom edge of the box corresponds to the 25% quartile and the top edge corresponds to the 75% quartile. The horizontal line is the median. *x* is the mean.

The hypothesis in the present study expected that clients who are coached by a human would have a greater working alliance than clients coached by a simulated AI. Contrary to expectations, this hypothesis is not supported. Clients coached by a human did not show a greater working alliance than clients coaching by a simulated AI. Instead, clients from both groups rated the working alliance with their coach in a similar range. An independent-samples t-test was run to determine if there were differences in working alliance between AI and human coaching groups. It was found that there was no statistically significant difference between human coaches (*M* = 74.50, SD = 7.25), *t*(50) = −0.71, *p* = 0.48 and AI coaches (*M* = 72.73, SD = 10.34). The mean value of the working alliance for both groups was moderately high, with the maximum score possible with the 12 items on a 7-point scale to be 84. This indicates a generally positive perception of the working alliance among respondents. Figure 4 shows a box plot showing a graphical representation of the distribution of working alliance comparing the two treatment groups.

An *a priori* power analysis was conducted using G*Power version 3.1.9.7 (Faul et al., 2007) to determine the minimum sample size required to test the study’s hypothesis pertaining to the working alliance clients developed with their coach. Results indicated the required sample size to achieve 80% power for detecting a medium effect, at a significance criterion of *α* = 0.05, was *N* = 102 for an independent samples t-test. Thus, the obtained sample size of *N* = 52 was less than the recommended sample size required to test this hypothesis.

### Qualitative findings

The qualitative findings were generated from responses of 13 individuals who were randomly assigned to the human coach treatment group and 14 individuals who were randomly assigned to the AI coach treatment group. These individuals were randomly selected to be interviewed about their experience and represent 52% of the participants in the study who received a coaching session.

The two concepts of the coaching process and working alliance show association to one another throughout the four themes that emerged from the qualitative analysis. The four themes indicate that the client’s connection with their coach existed within the unique circumstances of the study wherein the coach was a guide that used a variety of techniques to support the client to plan towards their goal. Table 2 shows the four themes and 15 sub-codes that emerged from the analysis with definitions of each that were uniquely generated from this study. The sub-codes are listed by theme in rank order from highest to lowest count along with the number and percentage of interview respondents who discussed that particular sub-code. Each of the four themes has a table with representative quotes from clients of both the human coaches and simulated AI coaches. Each participant has been given a pseudonym.

**Table 2:** Themes and sub-codes by treatment group.

Theme	Sub-code	Human Coach		AI Coach	
		*N*	%	*N*	%
*Client connection with coach* Client opinions about the coach’s intent and behaviors, responses that clients had when working with the coach, and descriptions of the dynamic between the client and the coach	*Rapport and relational tones* The quality of the interpersonal dynamic between the client and coach, reflecting both the depth of their bond and the nature of their communication	13	100.0%	13	92.9%
	*Affirmative emotional responses to coach connection* A client’s positive feelings and comfort levels that stemmed from their interactions and relational dynamics with the coach	11	84.6%	14	100.0%
	*Perceptions of Coach’s behavior and intent* A client’s understanding of a coach’s actions and underlying motivations, influenced by the coach’s demeanor, engagement level, and perceived authenticity during interactions	10	76.9%	11	78.6%
	*Adverse emotional responses to coach connection* A client’s negative or guarded feelings that arose from their interactions or perceived relational dynamics with the coach	7	53.8%	8	57.1%
	*Demographic factors of coach or client* The client’s awareness of specific attributes such as age, gender, race, or other demographic elements on the perceived bond and understanding between them and their coach	4	30.8%	7	50.0%
*Circumstances around the session* The range of expectations clients have coming into the session, the parameters around the coaching session, and client reactions to the technologies used in the session	*Client’s pre-session expectations* The beliefs a client held about the anticipated coaching session, influenced by initial perceptions of the upcoming interaction	10	76.9%	12	85.7%
	*Coaching session contextual parameters* The underlying assumptions and understandings that framed the environment of the coaching interaction influenced by past experiences	9	69.2%	11	78.6%
	*Technological interaction feedback* A client’s reactions and sentiments regarding the technological aspects of their coaching experience, encompassing both their comprehension of the platform and the emotional resonance elicited by its use	3	23.1%	14	100.0%
*Coaching techniques* Client comments regarding the coach’s use of questions, summarization, and offering of feedback as coaching techniques, plus the range of other techniques the coach employed in the session	*Questioning techniques employed by the coach* The specific strategies and methods the coach used to pose questions, aiming to provoke deeper introspection and clarity in the client’s responses	11	84.6%	13	92.9%
	*Feedback mechanisms adopted by the coach* The strategies used by the coach to provide insights, observations, and constructive critique to the client, enhancing self-awareness and guiding development	9	69.2%	14	100.0%
	*Other assorted coaching techniques* The varied methods employed by the coach, encompassing techniques ranging from discerning client priorities to adopting a more non-directive stance in the conversation	11	84.6%	12	85.7%
	*Coach’s summarization and clarification methods* The coach’s techniques of repeating back or rephrasing client statements, both to ensure mutual understanding and to aid in the client’s reflection process	8	61.5%	7	50.0%
*Client planning process* The client’s description of the process they went through with their coach to identify their goal and related action steps, along with what the client thought and did in relation to planning	*Journey from goal identification to action strategizing in the session* The process clients went through within a coaching session where they transitioned from recognizing their objectives to devising concrete strategies for achievement	13	100.0%	14	100.0%
	*Client’s chosen goals and tactical actions* The client’s explicitly stated objectives and selected deliberate steps or strategies they planned to take towards achieving the objectives that were identified during the coaching session	13	100.0%	14	100.0%
	*Client’s reflective insights during the planning conversation* The moments of self-awareness, revelations, and deeper thought processes experienced by the client while working through their goals and strategies during the session	12	92.3%	13	92.9%

#### Theme 1: client connection with coach

The theme *client connection with coach* is defined as client opinions about the coach’s intent and behaviors, responses that clients had when working with the coach, and descriptions of the dynamic between the client and the coach. This theme includes five sub-codes: (a) rapport and relational tones, (b) affirmative emotional responses to coach connection, (c) perceptions of coach’s behavior and intent, (d) adverse emotional responses to coach connection, and (e) demographic factors of coach or client (Table 3).

**Table 3:** Theme 1: client connection with coach.

Sub-code	Human coach Representative quotes	AI coach Representative quotes
Rapport and relational tones	“It’s like you are talking to professor but in the most lenient way, but still, you are maintaining that professionalism when you are talking. You’re not talking to just like a friend or talking to some executive of a company. So, the thing is, if you are talking to a friend, you are much too casual. Or when you are talking to some executive, you try to monitor yourself and look good ... I’d say it was not too professional or not too casual, something in a sweet spot. Like, I would describe it was like a good discussion, where you are trying to share something, and they are not directly giving you the answers ... The thing is, it did not matter what her expertise was. And it just brought out interest in me or solutions out of me.” (Shivam) “As we were going through the session the partnership got stronger, because [my coach] would be engaged and in tune with what I was saying, and start bringing out more of the value of what I was saying. So with more valuable insights, you start getting that credibility of, hey, this is actually working. And once you start getting that feedback, it kind of makes me just want to start going more soft now. I thought it was good. I like [my coach].” (Chris)	“I guess it was like moderate amount of being friendly, but also not overstepping. And also very insightful in a sense, like the key observations ... I felt like it was just much more insightful about myself. I learned more about myself than generally how my coaching sessions would go ... So I guess like, in that way, it was programmed very well. If that is how it is. It kind of pushed me to actually get the ideas out of me ... I just kept on talking. And I just got comfortable.” (Samira) “The coach asked great leading questions and asked about her curiosities with my life. I appreciated her sense of humor as well ... If I would tell a joke, then there would be... it’d be received. [My coach] would laugh and then it’d be very just conversational ... It’s interesting because sense of humor is so culture specific, too. It’s really interesting to see. You know, there probably would be another whole study on AI sense of humor.” (Huan)
Affirmative emotional responses to coach connection	“I never felt like I was talking to a complete stranger ... It just felt as if we knew each other ... because she was very nice. And she had a big smile all the time.” (Alessandra) “She seems very personable and caring. So I think with her demeanor allowed me to let my guard down a little bit to create the partnership. She’s very nice. She wasn’t gruff looking or anything.” (Chris)	“The best part is I found I feel more comfortable talking with [my coach] about some very sensitive topics that I would not talk with humans. Because I know [my coach], he’s an AI and he will not be so judgmental, and I do not need so much time to really get to know him. And we do not need to have so many small talks. And I can just cut to the chase and tell him what I’m really saying. I think that’s a very good part of talking with [my coach].” (Ling) “The session was very open and personal. It was quite enlightening and also I felt a sense of care and empathy from the coaching session ... I was not hesitating at all.” (Yuze)
Perceptions of Coach’s behavior and intent	“He seemed genuinely interested in me as an individual. It did not feel like this was a survey or anything like that. It felt, I suppose, felt like someone who truly cared about what was going on in my life. And I suppose I responded really well to that. He also made or commented, his insights on my personality and my circumstances, which made me feel very, for the lack of a better term, seen, made me feel very seen.” (Ethan) “We went through different discussions. I felt [my coach] was transparent, honest, and flexible to adapt to what I was trying to explain.” (Joaquin) “You just trust your coach, naturally to some way, especially that is a person who actually, you know, show the whole respect and non-judging and supportive, support setting or show things in a supportive, encouraging way.” (Zhi)	“I felt like the coach was my partner and a guide who was there in it with me, but also removed. I do not know if that makes sense. But was there as a partner with my best intentions at heart but also like disconnected in a healthy way. “(Joe) “We were both working toward the same goal, right? Its goal was to help me. So there was nothing antagonistic. It was collaborative.” (Rachel) “I think we both had a purpose. We knew what the purpose was. We acted on the purpose. Just very intentional.” (Aaliyah)
Adverse emotional responses to coach connection	“I think there was too much smiling ... I know there are people who react that way naturally. Let us say I’m like Hey, I’m doing physical therapy, and you are like Okay, but smiling, and that situation is not great. So I feel like there was just too much smiling ... So just, I do not know, connecting more to experiences.” (Amal) “The only part that was hindered was probably building that human connection. There was nothing that she and I bonded over the entire session ... There was no, *Oh, you are from Houston* ... I noticed that afterwards. I did not notice it while I was doing [the session]. It felt very transactional at the moment ... It felt very much like an exercise like I was going on a run with a buddy. We know what we are supposed to do here.” (Roberto)	“There were some long, awkward pauses at certain points to where I was reminded that it’s like a bot of some sorts, right? To where it kind of like... you feel like maybe you are starting to develop a relationship with this thing / person and tracking, and then they take this long pause. And it’s a little disarming, like, what’s going on? What’s happening? Did they listen? Is a timing out like, what’s going on? So that was kind of weird.” (Amanda) “I just realized that it could be an AI before I joined the meeting. And then I was kind of surprised. And I have to say I was kind of anxious at the very beginning. And I do not know how to react ... What kind of behaviors I should have towards interacting with an AI? Because I have not had this kind of coaching and really like a talking experience before. I was feeling kind of anxious and stressed for about 10 min and then we started to have some meaningful conversation, then I felt much better.” (Ling)
Demographic factors of coach or client	“I’m not an American. I’m an international student from Japan. But [my coach] seemed very American, like, typical American, I guess. She seemed very friendly and positive. She nodded. Her reactions were like a very big. So she felt like a very like American coach.” (Sakura) “She was a great listener. She seemed like very warm, like, she was listening to what I was saying... She would ask very good questions ... We built rapport pretty quickly. I might have also been because she was a woman. And so that was easier for me to relate to. But yeah, I think it was a good relationship.” (Gracia)	“[The coach] wasn’t like a black woman. It was a white guy who talked like a Bay Area bro. And that seemed to show a bit I guess like it showed its underbelly, I suppose ... I’d say [the voice and visualization] was a negative. Because I mean, I suppose everybody is coming to a session with experience with and that’s the problem of visualization is you cannot assume someone’s experience with that gender or ethnicity. I think I assumed a lack of empathy. And sort of an ego, I suppose there’s sort of an ego that comes with white male, Bay Area Bro-ness?” (Rachel) “As we went along, there was a sense of familiarity, even though like we had only been talking a little bit ... It got easier and easier to talk to her ... I think the fact that she was a woman made it easier, too. Like it was a woman’s voice. And also, as somebody who was older. So I felt like I could implicitly trust this person to talk to.” (Dahlia)

Representative quotes by treatment group.

#### Theme 2: circumstances around the session

The theme *circumstances around the session* is defined as the parameters around the coaching session, the range of expectations clients have coming into the session, and client reactions to the technologies used in the session. This theme includes three sub-codes: (a) client’s pre-session expectations, (b) coaching session contextual parameters, and (c) technological interaction feedback (Table 4).

**Table 4:** Theme 2: circumstances around the session.

Sub-code	Human Coach Representative Quotes	AI Coach Representative Quotes
Client’s pre-session expectations	“I had an intent ... I had a goal in mind. And so I wanted to get it through and I wanted to see what it was ... The anonymity of it is like, who cares, right? To open up about it, again, I think it was a combination of I thought there would be some anonymity about it because it’s just a one-time session and the intent.” (Roberto) “I’ve been following up with one specific life coach for almost 2 years now. And part of why I wanted to do this is to just see a different style. Just have another set of reference because I also had a goal in mind. And I’m like, maybe if I just see someone else, it would help me. Like, see what a different strategy would be. I came in really wanting to look for a different approach.” (Amal)	“Well, initially, before signing on, I feel like I was pretty ready to be vulnerable. And then I read the thing about the AI beforehand, and then I was just very curious. I think going to it like, what am I about to go into the Zoom? What is it what am I about to see when I into the Zoom call? So I think there was immense sense of curiosity, like an open mindedness in that way that I was like, I do not know what’s about to be on the other side of this, but I’m interested in what I find. So that kind of vulnerability, I think was maybe leading at that point, which wasn’t the kind of vulnerability I intended to go in with.” (John) “I think the way you set it up is really nice in terms of like, opening up the with that initial email and being like, oh okay, so it’s an AI [coaching] session. And as part of this research study, I think you are picking people who probably are more open to like the idea of you know, having an AI coaching session because I know I thought it was cool after like the first 5 min I was like, this is just cool.” (Yash)
Coaching session contextual parameters	“Because when I did have my coaching session through [the Institute] I kind of already knew what to expect, I knew the game plan. And I knew what to start talking about. And then I knew some of the questions - tell me more about this, what do you think that, and so forth. So it wasn’t a total surprise.” (Chris) “I do not think prior to my life coaching experience, I could have gone into this one [session] and come out with a clear goal and an action plan all in 45 min. The previous experience was absolutely necessary.” (Claire)	“But I think in general, when I think about executive coaching, I usually think, multi-session. And so I do not usually expect ... with any human, I do not usually expect to go very deep within the first session or two, because we are trying to fill it out. And so I guess I was expecting it to be more natural, like a natural, more natural flow like that typically tends to be a little bit more reserved start. And so I think to just like go in, it was different.” (Aaliyah) “I’m also a computer science student. And I have to say like, artificial intelligence is not something very new to me. That’s basically what I learn everyday. I’m in my last year of being a computer science student. But my background was kind of special too, because before I studied in a sociology and international development. So technically speaking, I do not have a very traditional computer science background like others. That’s why I wanted to join more of the leadership programs at [the university], so they can give me some suggestions ... I’m kind of like the unique one. That’s why I joined the program.” (Ling)
Technological interaction feedback	“I was struggling to pay attention … I needed to pay attention to what’s going on. So it’s not really [the coach’s] fault. But it [the AI media pieces in the prior email] was something that I think probably played into how I interpreted the session.” (Claire)	“I just got used to that, when she would laugh ... At first that really unnerved me. But then I kind of got used to that ... Those things that seemed manufactured ended up, like helping you buy into it. ... But those little things I mentioned, just kind of made me fall into this flow conversation. And it just got easier ... I kind of, I guess, accepted that this is what was going on. And I just went with it.” (Dahlia) “A lot of it, I think, was the tone of her voice. But she’d be like, mmmmhhhmmmm, and make little comments, or understanding that she was with you, tracking, listening, what have you. And when she talked she wasn’t just static, like she wasn’t just looking at you and just blinking. She was moving and it was very in sync with how... it looked like body language that would fit verbal cues. ... And she would take a second and she would turn her head and lean back and be thinking and saying things. So it felt very much like I do not know, it felt very human-like, if you will, to where her head expressions and as she was talking they would move and whatever with you. To where you felt kind of like she was thinking about it and she was trying to figure it out and she was driving with you.” (Amanda)

Representative quotes by treatment group.

#### Theme 3: coaching techniques

The theme *coaching techniques* is defined as client comments regarding the coach’s use of questions, summarization, and offering of feedback as coaching techniques, plus the range of other techniques the coach employed in the session. This theme includes four sub-codes: (a) questioning techniques employed by the coach, (b) feedback mechanisms adopted by the coach, (c) other assorted coaching techniques, and (d) coach’s summarization and clarification methods (Table 5).

**Table 5:** Theme 3: coaching techniques.

Sub-code	Human Coach Representative Quotes	AI Coach Representative Quotes
Questioning techniques employed by the coach	“And at the end, she asked me, she did not tell me. She never told me these were the steps and the actions. She asked me about them. But that was useful to help me remember what we had discussed. ... But maybe remember the idea is coming from you. Like that has a different... They resonate differently than if you hear them from someone else ... It always felt very natural, having a conversation with her. Also insightful. I would say the questions that she asked were very, very useful to just get me thinking.” (Alessandra) “Then we, together, and as I was explaining to him my goal, he was also asking me some questions. In that back and forth, I started reshaping my goal. Why reshaping my goal? Because I started realizing okay, probably I’m asking this and I should not try to focus on this ... he was not telling me what to do. And he was very, you know, he was ready to not answer my questions directly on what I would need to do.” (Joaquin)	“So I would say one of the [important] moments would be, you know, trying to get the guidance, and also the open ended questions that really helped me find out myself. Like what might be the issue. And how I can, you know, go about changing it in the future. So I think that, kind of, really helped me. And, kind of, impressed me. I did not expect that from a bot.” (Anika) “[My coach] basically asked me guided questions that would allow me to think about different perspectives as well. I think the main way that I think coaching works, and also is really how we gain new insights is, as I am like doing the thinking, but then they are guiding my thinking in a way with their questions.” (Huan)
Feedback mechanisms adopted by the coach	“I like that [my coach] follows up and picks up on ... red flags, like not red flags, but important things like [me] saying, hopefully I’ll do this ... She picked up on when we were pretty much restating our goals it picked up on, I was saying, I hope, hopefully, I’ll get this done. And [my coach] was like, hopefully, like, are you going to get it done? And I’m like, goodness, gracious, yes. And it makes such a huge difference.” (Amal) “I had a pretty difficult last semester. And then just the [other] people were pretty difficult. And then I eventually managed to get through that ... [My coach] said, I was very brave. And that made me very happy. Because I never realized that before. I never realized that I’m actually really great, so I was like, yay, she said that. I’m so happy she said that.” (Zhi)	“[My coach] making observations about my tone or behavior ... I think for the AI coach, like paying attention to my mannerisms very closely. We know what our own mannerisms are. And we, you know, might subconsciously try to correct them, but I think it’s hard. And so if other people call you out on them, it’s like, okay, people are paying attention ... I think there was a moment where I was expressing doubt about whether or not I would be able to accomplish my goals. And I appreciated that the AI coach picked up on that and sort of pushed me on it. That was the most important, probably the most, it’s the moment that I remember.” (Divya) “So I definitely think as it can be done perfectly by AI. It can pick up words, and then remind you: Okay, think deeper again, and make a deeper commitment. So I have no doubt about that, in terms of like picking up ... So for example, I remember clearly *I should*. And then I think the machine, or AI, or whoever it was said *okay, that does not sound like a big commitment*, right. *So you should or you will?* Sort of thing right, so it’s very specific.” (Yuze)
Other assorted coaching techniques	“I think she kept me on task – avoiding tangents, and then going off with the stories. So that was really good in terms of keeping me on task, talking about my goals ... Because she did not let me go off into my stories ... And that’s a really good coach or just did not care, right. But that was unique because most of the time I talk to coaches that’s what happens. We end up going off into tangents, right. There was none of that ... A lot of times people get interested in my stories, and then they let me finish my stories. And then they bring me back. I felt she was very abrupt in where she would stop the stories, which is great because it kept me on task ... And maybe this is just good coaching.” (Roberto) “I think [my coach] figured out pretty quickly that I’m a more visual person. And so the analogies really helped. That changed a lot for me. Thank you, [coach], because I’m able to see it in my head. So she was really good with analogies and that works for me. That’s just my brain thinks that way. So she was talking to me about like, neuro pathways ... And like, you have created these neuro pathways, and she said that it was like a highway. It’s well built. There’s paved roads, like this is how, what you have done your whole life. Like now you have to go find this like dirt road, it’s uncomfortable ... But if I do it long enough, maybe it becomes the street and then a highway.” (Gracia)	“The practice simulation, that we did would be also an important moment ... I think it was one of those things where it was like, just a little outside of my comfort zone enough to like, put me in that scenario, but in a space that was very low stakes. We agreed on action items that I can take, and the coach proposed a couple of simulations to do some quick practice for how I can do those action items.” (Chad) “I think they would sometimes give ideas as well. Yeah, but that was not as often. It was mostly just guided, but they would sometimes provide some ideas as well. ... And so what the coach, one of the ideas that the coach suggested was taking something that’s like a middle ground between having a basic calendar, or versus this intense productivity manager, and something that they were talking about was like a middle ground. And they basically suggested to think about what would be a middle ground for me.” (Huan)
Coach’s summarization and clarification methods	“The recaps were helpful as we were moving to a new topic, going from one topic to another ... I would say my version of the recaps and if I left something out, she would mention like if there was something else that was left out of my recap. I also liked that as we moved to different parts of the conversation, she would like bring up like what we have discussed so far, and to my own words, to help me like, kind of summarize where we are at.” (Gautam) “Maybe it’s just that I’m easy to please, but just the fact that he took the time and was able to summarize everything I was saying, which maybe my bar is just low, but that active listening really did it for me. The fact that he really took the time to internalize what I was saying. ... So that helped me to hear my own words.” (Ethan)	“And then being able to pick up on those themes and address them live and in conversation, and then being able to recall things from previously was interesting. Because sometimes, in conversations, I forget what people say person to person. It’s very normal. And so when she was recalling all that stuff, I was like, cool.” (Amanda) “I think that the almost like the speech patterns, there was a lot of like, like, almost repeating back what I said so like, it definitely, there was like the feeling of being like, heard or like being actively listened to. I think that was helpful.” (Chad)

Representative quotes by treatment group.

#### Theme 4: client planning process

The theme *client planning process* is defined as the client’s description of the process they went through with their coach to identify their goal and related action steps, along with what the client thought and did in relation to planning. This theme includes three sub-codes: (a) journey from goal identification to action strategizing in the session, (b) client’s chosen goals and tactical actions, and (c) client’s reflective insights during the planning conversation (Table 6).

**Table 6:** Theme 4: client planning process.

Sub-code	Human Coach Representative Quotes	AI Coach Representative Quotes
Journey from goal identification to action strategizing in the session	“To make progress I think at some point, after defining the goals and after trying to be very clear, or understand what I wanted, [my coach] was very useful on that side. And if you clarify your idea, that’s progress. I think that that’s the most important. Next steps, he also made me reflect out loud about what are the next steps, trying to understand my goals and how I can explore my capabilities.” (Joaquin) “I think mainly the process, which was most important part, the approach ... I think there’s not like one big important part. But I think everything contributed to the main thing. That’s how I would like to put it, because it was like in a series of actions and step by step, right. Even if like one step was missed, I would not be ending up in the last moment. Like it would have been turned out in a different way .... Mainly finding out my short-term goal and my current needs was something like a starting point ... We spent the first 10 min trying to understand what my goal was ... Then we went through, she asked me questions regarding how am I achieving this goal, what I’ve done so far, where do I feel I’m lacking? ... Then she asked me, I think she never gave like a direct answer or like solutions, instead helped me find out my own solutions. Figure out what and where it is going wrong. This is I think this is something which kind of helped in a way to reiterate what I was doing and how I was doing, how effectively I was doing that. And that was like, the whole goal of the thing, it helped me find out and check where I progress in that thing.” (Shivam)	“We walked through my value systems and he encouraged me to think about ... what are my core values. And I explained what my core values are ... And by probing me a little bit further on that it made me realize that I could use my passion for improving relationships ... and use that as a way to improve, to guide my goal of wanting to be more productive at work. Rather than just coming up with step one: wake up on time, step two: use an app to log my hours ... Which are what I came up with. It helped me think about the big picture: how can I use my value system to actually achieve those more superficial tasks that are to be achieved in order to achieve my goal? “(Yash) “I guess I tried to deflect at that point, generally, but that is my tendency. I was like, *Yeah, I’m thinking about it*. But this time, [my coach] was more like, *okay, but I’m going to push you some more*. And I want you to see exactly this, exactly that. So I think that was probably the first time I was really pushed to a corner where I have to commit to something. Or say something that I’m going to write this down in my goal statement, modify it, or add it, and do that. Exactly this ... That is something definitely I realized that I really had to commit to something specific. Yeah, I’m going to do this checklist. That was something new.” (Samira)
Client’s chosen goals and tactical actions	“I appreciated that we set clear steps towards the goal I wanted to work towards. Additionally, the coach towards the end of the session prompted me to reiterate what I learned about myself in the session, as well as summarize the steps to take to achieve my goal. This is pushing me to commit more to what I want to do.” (Amal) “When we identified the main goal that I wanted to work with, I wasn’t sure of what were the steps that I needed to do. But continuing the conversation with my coach, we started to talk and I started to identify [the actions] ... My goal is more related with my professional development, because I do not know, sometimes graduate course, or graduate schools can be kind of stressful ... And now I can identify that the possible steps that I can do are these ones.” (Mateo)	“I am going to evaluate every ask of my time and where necessary request alternatives to have less stacked back-to-back on my plate ... It’s been really, I’ve been very thoughtful about thinking through as I get requests, and as I get asks, like, is it something that I can ask for a rescheduling for? Or is it something that I need to prioritize right this at this moment? Or is it something that can be pushed to later? And so I think it just made me be more thoughtful about as I planned the time, what that looks like, and what makes the most sense for me.” (Aaliyah) “The appropriate steps, or the follow up steps, were very appropriate and very doable ... And if you think that the people would be willing and open to explaining why they do what they do, then you know, why not do that? So I thought that was a moment that kind of sticks out just because it’s so simple, like a low hanging fruit type of activity to do.” (Joe)
Client’s reflective insights during the planning conversation	“Sometimes [it] can be uncomfortable to discover some insights. I did talk to [my coach] about some fears of why I need to do something but I’m not doing it ... It does help like talking it out. Like I wasn’t very surprised with my answers to the probing. Like I knew that, but I think it helps speaking it out loud ... Also, I think that led us to think of other ways to measure progress in terms of weight loss. Like you might not see pounds go down, but you might be able to see, like if you are measuring yourself with measuring tape, you might be able to see loss there in centimeters ... So that stood out, figuring out on using different ways to measure progress ... We constructed why there are certain things I do not do in terms of weighing myself. It’s kind of like that fear of seeing no change going day to day.” (Gautam) “I’m looking to go into to the public sector, and working social policies, mostly, like anti-poverty programs. But there’s one thing that I’ve always felt sort of uneasy about. Like if I’ve never experienced those things myself, then who am I to represent people that I do not even fully understand because I’ve never gone through what they have lived all their lives ... [My coach helped me think of] maybe asking other people that are doing it, can give a very good insight as to how they address that part of the job ... And that really got me thinking ... I thought a lot about the answer to that question, and it made me realize lots of things.” (Alessandra)	“I think [my coach] forced me to reevaluate, like my motivation and my perspective on the things that I am trying to do in my life” (Dahlia) “What I think was the big aha moment for me, which was crazy, was that it kind of strayed away from the conversation that I was trying to have about productivity at work. And that’s my original goal. And then it got me to think and dive deeper into my emotional state and how I’ve been throughout the years. And [my coach] picked up on when I figured out that this is something I’ve been struggling with for a long time. And there’s specific values or behavior characteristics that I need to work through that go beyond my goal itself ... I need to dive deeper than just my superficial goal of like, how can I increase the amount of time I’m being productive and you dive deeper into thinking about how I’ve operated as a person where I have not been able to find the right work life balance, the things that I need to work on, particularly with discipline and communication and responsibility, and in holding myself accountable.” (Yash)

Representative quotes by treatment group.

### Believability index

The believability index was constructed as a manipulation check in this WOz experiment. As commonly found in social psychology and human-computer interaction research, a manipulation check assessed if and to what extent participants believed the deception in the study. In order to examine the relationship between the three items in the believability index, Pearson’s correlations were conducted. It was found that there is statistically significant correlation between qualitative coding and the AI belief question, *r*(52) = 0.69, *p* = 0.000. The correlation between qualitative coding and the human belief question was statistically significant, r(52) = −0.55, *p* = 0.000. Finally, the correlation between AI belief question and human belief question was also statistically significant, *r*(52) = −0.71, *p* = 0.000. Given that these correlations were statistically significant, relationships among them were in the expected direction, and that they were moderate in magnitude, it provided the conditions needed to use the three items to create a believability index. There were two believability indices, one for whether participants believed they were interacting with an AI coach and another for whether participants believed they were interacting with a human coach.

To determine whether participants assigned to the AI condition “believed” that they were interacting with an AI coach an independent samples *t*-test was conducted. Three items were combined – the researcher’s code of qualitative answers, the answer to the AI belief survey question, and the reverse-coded answer to the human belief survey question. The Cronbach’s alpha for the three items that form the Believability Index for AI is 0.890. Using a believability index that was coded for belief in interacting with an AI, a statistically significant difference was found between participants in the AI (*M* = 14.19, SD = 2.88) and human conditions (*M* = 5.12, SD = 2.79), *t*(50) = 11.53, *p* = 0.000. Results showed that participants in the AI condition believed they were interacting with an AI coach, whereas participants in the human condition did not believe they were interacting with an AI coach (Table 7).

**Table 7:** Difference between AI and human coaching groups on belief in interacting with AI.

	AI coach group		Human coach group				
	M	SD	M	SD	df	*t*	*p*
Interacting with AI	14.19	2.88	5.12	2.79	50	11.53	0.000

Conversely, to determine whether participants assigned to the human condition “believed” that they were interacting with a human an independent samples t-test was conducted. Three items were combined—the researcher’s code of qualitative answers, the answer to the human belief survey question, and the reverse-coded answer to the AI belief survey question. The Cronbach’s alpha for the three items that form the Believability Index for Human is 0.843. It was found that using a believability index that coded for belief in interacting with a human, a statistically significant difference was found between participants in the AI (*M* = 6.81, SD = 2.88) and human conditions (*M* = 15.88, SD = 2.79), *t*(50) = 11.53, *p* = 0.000. Results showed that participants in the human coach condition believed they were interacting with a human and participants in the AI condition did not believe they were interacting with a human coach (Table 8). These analyses provide evidence for the validity and credibility of both the AI and human coach conditions.

**Table 8:** Difference between AI and human coaching groups on belief in interacting with a human.

	AI coach group		Human coach group				
	*M*	SD	*M*	SD	df	*t*	*p*
Interacting with Human	6.81	2.88	15.88	2.79	50	11.53	0.000

## Discussion

The mixed methods RCT compared client experiences of being coached by a professional human coach or a simulated autonomous AI coach. The study sought to understand the quality of the relationship that the participants, or clients, built with their coach during a one-time 60-min coaching session. As well, the study examined the coaching process and perceptions of how the session unfolded for individuals in both treatment groups. The results show that both treatment groups built a moderately high working alliance with their coach, whether it be the professional human coach or the simulated AI coach. The qualitative findings illustrate important aspects of the working alliance and how it was built during the session.

The initial hypothesis—that professional human coaches would form stronger working alliances with clients than simulated AI coaches—was logical, both from a practical standpoint and supported by previous research in AI coaching and related modalities. However, this hypothesis was not supported. Clients built similar moderately high-quality relationships whether they thought their coach was a human or AI. More specifically, the participants in this study did build a relationship with their simulated AI coach. These results offer several implications for coaching research and practice.

### Client perspectives of AI coaches

The majority of working alliance themes from the qualitative data were described in similar ways across the two coaching treatments. However, there were a few of areas that had differentiation that are worthwhile to highlight. First, a key difference between the two groups was that participants in the professional human coach group provided demographic-based feedback about their coach less frequently and with fewer details. Those in the simulated AI treatment group shared demographic-based feedback about their coach more often and were much more open and forthcoming with their preferences. This could mean that the demographics (e.g., age, race, gender) of AI coaches and the way those are portrayed through the coaching experience (e.g., visual, audio, backstory references) does matter to clients. The ways participants described their coaches was akin to the three-part construct of AI anthropomorphism with physical, personality, and emotional traits (Epley, 2018; Epley et al., 2007). As for the visual interface, in a similar study Kang and Kang (2023) found that this did affect the self-disclosure and companionship of participants in a counseling intake session. Chaves and Gerosa (2020) argued that to avoid participant dissatisfaction, the optimal design of an AI’s social characteristics would take into consideration the expectations of participants. Similarly, Franco et al. (2021) suggested that client ability to personalize their avatar coach could result in higher affective bond and better outcome results. Alabed et al. (2022) went so far as to propose a theoretical framework to connect AI anthropomorphism with its effect on participant self-congruence and self-AI integration with consequences including a person’s emotional connection with the AI. Participants in the present study said that in the future they would want to choose the features of their AI coach to enhance their connection, comfortability, and openness with their AI coach.

The second key difference between the two treatment groups was that even though both felt comfortable talking openly with their coach, those in the simulated AI coach group were pleasantly surprised at their positive emotional response to the AI. The main difference was that those in the AI group emphasized that they did not expect to be so vulnerable with their coach and were pleased with the non-judgmental atmosphere (Ellis-Brush, 2021; Nass and Moon, 2000). Remember that the participants in this study had previous leadership coaching experiences from a similar pool of trained coaches. With this in mind, several participants who were coached by the simulated AI stated that they felt safer with their AI coach than with their previous human coach. This aligns with literature from the counseling field, where it has been found that individuals who have post-traumatic stress disorder prefer to partner with digital avatars because they feel free to be more vulnerable with avatars than with a visible human therapist who is perceived to be judging them (Lucas et al., 2014; Seitz et al., 2022).

Individuals who were coached by the simulated AI shared that they did not feel it was necessary to use the impression management behaviors that they would normally use in human-to-human conversation. To maintain a desired image with others, impression management behaviors, whether intentional or unconscious, are intended to shape how people are seen by others (Blunden and Brodsky, 2024). As individuals use less impression management behaviors, they are more of their true selves. To describe this further, McFarland et al. (2023) created a contextual framework for understanding impression management and identified contextual influences on impression motivation (i.e., public vs. private, situation stakes, evaluation event proximity, target status) and contextual influences on impression behavior (i.e., permanence, verifiability, anonymity, synchronicity). In this study, those in the simulated AI coach group were in a low-stakes, private context and with a low-status target. The information clients shared synchronously in the session had low permanence with hardly any documentation of it with low verifiability by the primary researcher or professional coaches involved. This indicates that the perception of using an AI coach may create an environment that has a lower motivation to use impression management behaviors compared to human coaches, which could result in higher psychological safety (Edmondson and Lei, 2014).

### Relationship building with AI coaches

During the debrief interviews, participants shared their experience with how they built a working alliance with their AI coach. First, participants said that they entered the coaching session committed to gaining value from it. Individuals were ready to put in the effort required to gain insights about their topic and brainstorm action items to get them closer towards their goal. As other research has shown, client commitment to the coaching experience and readiness for change are active ingredients in what can make coaching valuable (de Haan, 2019; McKenna and Davis, 2009). Second, participants said they were open to the coaching experience, and were curious to observe how it would unfold when being coached by the AI. Upon receiving the email notification that they would be coached by an AI, some participants reported becoming even more open than they were initially. Böckle et al. (2021) from the human-computer interaction field found that individuals with the Big Five personality trait of open-mindedness had correlative levels of trust in AI interfaces. For the participants in the present study, they were open-minded to engage with the AI coach, and yet they did not let that curiosity distract them.

Third, participants said they strived to remain fully engaged in the coaching session, concentrating on their chosen subject matter rather than being distracted by the novelty of the situation. When first starting the conversation, some clients said that it took a few minutes to accept that they were speaking with an AI. After becoming comfortable with the avatar modality and conversational interaction, clients said that they were no longer distracted by the AI interface. Being present is an important competency that is emphasized for coaches to do well in their roles (Erdös et al., 2021; Maltbia et al., 2011) and in this study the clients shared that it was important for them to be present as well. Alongside remaining present, fourthly in how clients built a relationship with their AI coach, they were responsive to the interactions their AI coach offered to them within the coaching conversation. Participants noted that the AI coaches used a variety of techniques, such as asking questions, offering feedback, and sharing summaries, to support client reflection and growth. The participants responded to the AI coach’s prompts, fostering a collaborative dynamic that evolved throughout each 60-min session.

### Limitations

Two notable limitations within this study are relevant to highlight. First, this study was designed with a one-time coaching session rather than a longer coaching engagement consisting of several sessions occurring over a longer time period. Much of the previous coaching research analyzes circumstances where the coaches and clients work together for more than one session with a partnership that is built over time. Recent findings indicate that working alliance does not change much over the course of a coaching relationship (de Haan et al., 2020; de Haan and Nilsson, 2023; Stiles et al., 2015), however the studies included in these analyses are not typically designed with only one coaching session. It would be interesting to compare the results of this study to other studies designed with one coaching session that have a rigorous methodological design.

Second, another limitation is the historical time period within which this study was conducted – during the generative AI hype of 2023 (McKinsey & Company, 2023b). This was a unique influence at this point in time that the researcher assumes made it easier for participants to believe they were being coached by a real AI rather than a simulated one. It made it possible to examine the use of a future-state version of AI rather than a more elementary version that is currently available today. With this, it is tough to generalize the results of this study to other AI research situations, particularly in those published prior to 2022, yet also in the future. For future replication, it would be valuable to conduct a similar study in a few years to examine how client perceptions evolve alongside advancements in AI.

### Future research

Two main ideas for future research include (1) investigating the effectiveness of various AI coaching models, and (2) conducting additional studies to assess working alliance when clients use an AI coach. First, to further explore expert systems of human coaching applied via AI, additional research could be conducted that aligns to any of the four models presented earlier in this paper (Clutterbuck, 2022; Duhan et al., 2023; Graßmann and Schermuly, 2020; Terblanche, 2020). Given that the technology is not advanced enough at this time to fully embody a human coach, researchers could begin exploration with the current abilities of AI, as Terblanche has done with designing Coach Vici. With the current limitations of AI, it is likely more reasonable for AI coaches to have specific areas of foci to address specific topics, rather than be broadly focused on a wide variety of potential client session topics like a human coach could (Terblanche, 2020). Beyond studying how expert systems of human coaching work with AI, alternative models could be explored to understand if and how coaching techniques might need to be adapted to meet client expectations when delivered via AI. Future research will tell if what works well with human coaches also works well with AI coaches, or if different and new models of AI coaching are necessary to create (Herbener et al., 2024). Incorporating the aforementioned models into future research endeavors provides a structured approach to dissect the intricacies of AI-facilitated coaching. This comprehensive exploration would not only refine current models but could potentially lead to the development of innovative frameworks tailored for AI coaching efficacy that depart from the human-based expert models. The exploration of specific coaching domains, as suggested by Duhan et al. (2023), could be particularly insightful in identifying which coaching strategies are most amenable to AI translation. Bachkirova and Kemp (2024) recently assessed simple types of organizational coaching and identified several elements of the coaching process that could be augmented by AI. In order to further build on the standard coaching models, cross-disciplinary research drawing from cognitive science and human-computer interaction could enrich our understanding of the nuanced ways in which AI can integrate with human coaching techniques, as posited by Clutterbuck (2022). With the rapid advancement and adoption of generative AI tools by the population at large (Pew Research Center, 2023), the field anticipates the amount of AI coaching research to increase alongside it (Tavis and Woodward, 2024).

A second idea for future research is to expand the number and type of research studies focused on working alliance between AI coaches and human clients. The new research could focus on different client populations, coaching styles, or client goals. The coaching field could borrow from the human-computer interaction field with its varied ways to conduct research with new technologies – hosting co-design workshops, facilitating interviews or focus groups, gathering feedback through prototype testing, implementing WOz experiments, and assessing established commercial systems (Clark et al., 2019). These research methods can be conducted at the appropriate stage of technological innovation systems lifecycle as new coaching AIs are established (Markard, 2020). Research could investigate the nuanced psychological cues that AI should emulate to establish trust and openness with clients. Considering the complexities of human emotion and self-disclosure, studies could examine the extent to which AI can replicate the empathetic and nonjudgmental stance of human coaches and client response to that (Dai et al., 2024; Pataranutaporn et al., 2023; Shao, 2023; Yalçın, 2020). Research could evaluate the client’s psychological safety when interacting with AI, measuring the depth of rapport compared to that with human coaches. Investigations might also incorporate elements from the fields of artificial empathy (Dial, 2018; Morrow et al., 2023) and relational AI (Anisha et al., 2024; Mariani et al., 2023) to design coaching systems that clients find genuinely supportive and engaging. Additionally, longitudinal studies could illuminate the development of working alliances over time between AI coaches and clients, offering a dynamic view of relational growth.

Other ideas for future research include: (a) exploring hybrid intelligence systems, or the partnership between artificial and human intelligence, that are attuned to the field of professional coaching (Akata et al., 2020; Dellermann et al., 2019; Jarrahi et al., 2022), (b) assessing personalization of AI coaches with matching or design criteria that matter to different client profiles (Kang and Kang, 2023; Nißen et al., 2022), and (c) exploring client perceptions of partnering with AI coaches at different phases throughout the coaching process (Mitchell et al., 2021).

### Practical implications

The implications of this study for the coaching industry are clear: the longstanding belief that human coaches are irreplaceable is challenged by the findings in this study from clients who are willing to engage with AI coaches. Current AI technology, despite its limitations, has already proven effective in areas like goal facilitation, as evidenced by Terblanche et al. (2022b). Moreover, public opinion and consumption of AI is increasing with McKinsey, the global consulting firm, having estimated that AI, including generative AI and other forms of AI like machine learning, could unlock up to $25.6 trillion of value for the global economy (McKinsey & Company, 2023a). With the expansion of AI there is a need for professional coaching associations, coaching educators, professional coaches, and third-party coaching providers to embrace innovation with AI.

Professional coaching associations should continue to shift their perspective to proactively update their standards, foster partnerships with AI developers, and provide guidance on AI-related ethics and practices. A few years ago, the ICF established the artificial intelligence coaching standards work group, and out of this group in June 2024 the Artificial Intelligence (AI) Coaching Framework and Standard was published (International Coaching Federation, 2024). The framework offers guidance for developing responsible AI coaching systems and aids clients in identifying systems that align with these best practices. Coaching educators and training organizations should design and implement curricula that incorporate AI literacy and comprehensive ethics training, offer continuous professional development and specialized certifications in AI, and develop collaborative models to demonstrate how AI and human coaches can effectively work together, thereby differentiating themselves in the market (Tavis and Woodward, 2024).

Independent professional coaches should proactively educate themselves about AI advancements and integrate AI tools into their practice to enhance coaching methodologies, improve client experiences, and streamline business operations, thereby remaining competitive and responsive to the evolving demands of the coaching industry. Even though independent coaches can gain benefit from using AI, Diller et al. (2024) found that when coaches were asked about AI coaching, they felt threatened by it, which led to lower curiosity and a more negative opinion of AI. In the same survey, 57% of respondents said they did not consider AI as being able to deliver coaching.

Third-party coaching providers can continue to strategically integrate AI into their offerings to meet evolving client demands, invest in research and development of AI coaching tools, and develop diverse AI coaching solutions to address various client needs and preferences. This can help third-party coaching providers maintain a competitive edge, while integrating in to larger, systemic organizational learning strategies (Tavis and Woodward, 2024). As AI is integrated into coaching practices, ensuring data privacy and security is crucial for preserving client confidentiality, complying with evolving regulations, and maintaining trust, requiring all stakeholders to be informed about cybersecurity developments and apply ethical data governance while protecting sensitive information (DiGirolamo, 2024; Diller, 2024).

## Conclusion

The working alliance—a key factor in successful coaching experiences—must be re-evaluated in the context of human-AI interactions. This research represents a critical advancement in the nascent intersection of coaching and AI, addressing a gap in the literature where the concept of a simulated autonomous AI coach had not been previously examined. The study’s results delivered compelling results: clients formed a good working relationship with their coach – whether they perceived their coach to be an AI or a human – and as strikingly equivalent, challenging preconceived notions of AI’s lacking efficacy in coaching contexts. AI coaches of the future, when they are designed with the expert model of human coaching, can build solid working relationships with clients. Participant stories in the debrief interviews illuminated the reasons why and how the coaches resonated with them – highlighting the unique contributions of this mixed methods study. These insights point to a potential paradigm shift in coaching practices, where AI’s role may be far more integral and effective than previously imagined.

## Data Availability

The raw data supporting the conclusions of this article will be made available by the authors, without undue reservation.
